# Citri Sarcodactylis Fructus Alleviates LPS‐Induced Acute Lung Injury by Inhibiting Inflammation and Inflammasome Activation

**DOI:** 10.1002/fsn3.70881

**Published:** 2025-09-01

**Authors:** Zhuohui Luo, Zaibin Xu, Kongyan Wang, Huiyu Hu, Jiawen Huang

**Affiliations:** ^1^ Hainan Pharmaceutical Research and Development Science Park Hainan Medical University Haikou China; ^2^ Science and Technology Innovation Center Guangzhou University of Chinese Medicine Guangzhou China

**Keywords:** acute lung injury, citri sarcodactylis fructus, molecular docking, molecular dynamics simulation, NF‐κB signaling, NLRP3 inflammasome

## Abstract

Citri Sarcodactylis Fructus (CSF), an ornamental and edible fruit belonging to the genus *Citrus* in the *Rutaceae* family, has been shown to exhibit anti‐inflammatory, antioxidant, and anti‐obesity effects. However, its impact on the progression of acute lung injury (ALI) remains unclear. In this study, CSF significantly alleviated LPS‐induced pulmonary edema in ALI mice and improved oxidative stress markers, as evidenced by reduced serum malondialdehyde (MDA) levels and increased serum superoxide dismutase (SOD) levels. It also decreased macrophage and neutrophil counts as well as protein levels in bronchoalveolar lavage fluid (BALF), and inhibited inflammatory responses‐specifically, it reduced the protein and gene expression of IL1β, IL6, and TNF‐α in lung tissues, downregulated the mRNA levels of chemokines, and suppressed LPS‐induced activation of NF‐κB signaling. Moreover, CSF significantly reduced NLRP3 inflammasome activation. The components of CSF exhibit high binding activity and stability to NF‐κB p65 or NLRP3, along with low binding free energy, within their respective binding pockets. This study reveals that CSF alleviates LPS‐induced ALI by inhibiting NF‐κB signaling and NLRP3 inflammasome activation‐mediated inflammatory response, thereby providing a scientific basis for the utilization of CSF resources.

## Graphical abstract

Citri Sarcodactylis Fructus (CSF) has been shown to exhibit anti‐inflammatory, antioxidant, and anti‐obesity effects. However, its impact on the progression of acute lung injury (ALI) remains unclear. This study reveals that CSF alleviates LPS‐induced ALI by inhibiting NF‐κB signaling and NLRP3 inflammasome activation‐mediated inflammatory response, thereby providing a scientific basis for the utilization of CSF resources.

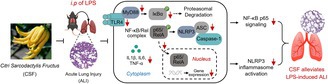

**Abbreviations**
ALI: acute lung injury
ARDS: acute respiratory distress syndrome
ASC: apoptosis‐associated speck‐like protein containing a CARD
BALF: bronchoalveolar lavage fluid
Caspase‐1: cysteinyl aspartate specific proteinase‐1
CSF: Citri Sarcodactylis Fructus
DAMPs: damage‐related molecular patterns
Dex: dexamethasone
E_b_
: binding energy
E_ele_
: electrostatic energy
E_ps_
: polar solvation energy
E_SA_
: SASA energy
E_vd_
: van der Waals energy
GO: Gene Ontology
H&E: hematoxylin and eosin
KEGG: Kyoto Encyclopedia of Genes and Genomes
LBP: lipopolysaccharide‐binding proteins
LPS: lipopolysaccharides
MD: molecular dynamics
MDA: malondialdehyde
NLRP3: NOD‐like receptor thermal protein domain associated protein 3
PAMPs: pathogen‐related molecular patterns
PPI: protein–protein interactions
Rg: radius of gyration
RMSD: root mean square deviation
RMSF: root mean square fluctuation
SASA: solvent‐accessible surface area
SOD: superoxide dismutase
TCMSP: Traditional Chinese Medicine Systems Pharmacology Database and Analysis platform
TLR4: toll‐like receptor 4

## 1 Introduction

Acute lung injury (ALI) is a serious respiratory disorder, primarily characterized by hypoxemia and respiratory distress, with an extremely high morbidity and mortality rate, seriously threatening the lives of critically ill patients and affecting their quality of life (Rubenfeld et al. 2005; Fan et al. 2005). Systemic and local inflammatory responses are key underlying mechanisms in the onset and progression of ALI. While drugs such as glucocorticoids effectively regulate inflammatory response to prevent and treat ALI, they are also associated with side effects including immunosuppression and secondary infections (Cain and Cidlowski 2017). Therefore, exploring novel therapeutic or functional interventions remains necessary.

Acute inflammation, an important manifestation of tissue damage, is closely associated with the pathogenesis of ALI. During the development and progression of ALI, activation of NF‐κB induces the expression of a series of pro‐inflammatory cytokines and chemokines (Hu et al. 2016), thereby triggering the recruitment and activation of inflammatory cells and exacerbating pulmonary inflammatory responses (Vermeersch et al. 2024). Lipopolysaccharides (LPS), an endotoxin, interact with Toll‐like receptor 4 (TLR4) on the membrane surface of host cells, activate NF‐κB to initiate cascade amplification of the inflammatory response (Zhao et al. 2024), and thereby exacerbate lung tissue injury, causing more severe damage to the normal structure and function of the lungs (Deng et al. 2024). Moreover, NOD‐like receptor thermal protein domain associated protein 3 (NLRP3), as a type of pattern recognition receptor, binds to apoptosis‐associated speck‐like protein containing a CARD (ASC) by recognizing damage‐related molecular patterns (DAMPs) and pathogen‐related molecular patterns (PAMPs) (Yehya et al. 2024). It then recruits and activates cysteinyl aspartate specific proteinase‐1 (Caspase‐1), induces cellular pyroptosis, promotes the maturation and release of pro‐inflammatory cytokines IL1β and IL18 (Xu Hu, et al. 2024), and exacerbates inflammatory damage to lung tissues (Yang et al. 2024). Thus, reducing the inflammatory response is an effective way to alleviate ALI.

Citri Sarcodactylis Fructus (CSF), mainly produced in Guangdong and other parts of China, is an ornamental and edible fruit of *Citrus medica* L. var. *sarcodactylis Swingle* of the Rutaceae family. It has a unique fragrance and contains various active ingredients, especially flavonoids (Deng et al. 2023), exhibiting multiple biological activities such as antioxidant, anti‐inflammatory, and antibacterial properties (Kim et al. 2013). In traditional Chinese medicine (TCM) theory, CSF enters the Lung meridian and has the effects of drying dampness, resolving phlegm, regulating qi, and relieving chest stuffiness. Its traditional use in treating respiratory diseases provides a theoretical basis for the treatment of ALI (Hu and Jin 2002). Modern pharmacological studies have shown that bergapten and flavonoids in CSF can reduce the release of proinflammatory factors to protect lung tissue (Dong et al. 2020; Liao et al. 2024; Guo et al. 2019). In addition, limonene in its volatile oil can alleviate airway spasm and promote sputum excretion; animal experiments have confirmed that it reduces eosinophil infiltration in asthma models, demonstrating anti‐inflammatory potential (Chi et al. 2013). Given that ALI is driven by excessive inflammatory responses, we hypothesized that CSF, with its inherent anti‐inflammatory capacity, could ameliorate LPS‐induced ALI‐potentially via mechanisms similar to the anti‐inflammatory drug Dexamethasone (Dex), which inhibits inflammation by regulating transcription factors (Zhao et al. 2025; Li et al. 2023). However, the specific effects and underlying mechanisms of CSF in ALI remain unclear. To test this hypothesis, the present study used an LPS‐induced ALI model in C57BL/6 mice combined with systemic pharmacology to investigate the functional roles and potential mechanisms of CSF in ALI.

## 2 Materials and Methods

### 2.1 Chemicals and Reagents

Lipopolysaccharides (LPS) were purchased from Sigma‐Aldrich Co. (Shanghai, China). Dex was purchased from Aladdin (Aladdin, Shanghai, China). Unless otherwise mentioned, reagents were obtained from Sigma‐Aldrich.

### 2.2 CSF Sample Preparation

CSF (Batch No.: 230701411) was purchased from KANGMEI Co. Ltd. (Chengdu, China). The MPNS database (http://mpns.kew.org) was used to match the plant name. The medicinal herbs (500.5 g) were soaked in 10‐fold water for 1 h, then refluxed for 1 h, with Filtrate 1. After that, the residue was refluxed for 1 h with 10‐fold water again, with Filtrate 2. Finally, Filtrate 1 and Filtrate 2 were combined, concentrated, and freeze‐dried, with a yield of 39.81%. The CSF sample was stored at −20°C for further experiments.

### 2.3 UPLC‐MS/MS Analysis of CSF

To identify the chemical composition of CSF, UPLC‐MS/MS analysis was conducted as previously described (Xu, Li, et al. 2024). Briefly, CSF samples were analyzed by the following chromatographic conditions: ACQUITY UPLC HSS T3 column (100 mm × 2.1 mm, 1.8 μm) temperature 45°C at a flow rate of 0.35 mL/min with water containing 0.1% formic acid (A) and acetonitrile (B): (0–2 min, 95% A; 2–4 min, 95%–70% A; 4–8 min, 70%–50% A; 8–10 min, 50%–20% A; 10–14 min, 20%–0% A; 14–15 min, 0% A; 15–15.1 min, 0%–95% A; 15.1–16 min, 95% A), and the injection volume was 5 μL and scanning range of PDA was 210–400 nm. The mass spectrometry conditions were as follows: the ion source was HESI; the sample mass spectrometry signals were collected using positive and negative ion scanning modes. The data collection mode was DDA and the scanning method was Full MS/dd‐MS2 (TOP 8). Compound identification was based on accurate mass, secondary fragments, and isotopic distribution, with qualitative analysis performed using the LuMet‐TCM database (OeBiotech Co. Ltd., Shanghai, China), which has a total library capacity of over 5000 reference standards.

### 2.4 Animals and Animal Experiments

8‐weeks‐old C57BL/6J male mice, provided by Guangdong Medical Laboratory Animal Center, were acclimatized in an SPF‐level environment for a week, with a temperature of 24°C ± 2°C and a 12/12 light/dark cycle. Subsequently, mice were randomly divided into five groups of six mice each, including groups Con, LPS, LPS + Dex 1 mg/kg, LPS + CSF 50 mg/kg, and LPS + CSF 100 mg/kg. After 1 week of oral gavage with CSF. Mice were injected intraperitoneally with LPS (5 mg/kg) to induce ALI, and 24 h later, mice were sacrificed to collect serum, bronchoalveolar lavage fluid (BALF), and lung tissue for detection. All experimental procedures were approved by the Ethics Committee of Guangzhou University of Chinese Medicine (No. 20240912005).

### 2.5 Lung Wet/Dry (W/D) Ratios

Lung tissue samples were weighed to obtain the wet weight at the time of collection, and then placed in an oven at 80°C for 48 h and weighed again to obtain the dry weight. The wet/dry (W/D) ratios were measured to evaluate lung tissue edema.

### 2.6 Measurement of MDA and SOD

Malondialdehyde (MDA) and superoxide dismutase (SOD) were detected in serum using commercial kits (Nanjing Jiancheng Bioengineering Institute, China) following the manufacturer's instructions.

### 2.7 Histopathological Observation

Lung tissues were fixed in 4% paraformaldehyde for 24 h, dehydrated, embedded in paraffin, and then sliced into 5 μm sections. The sections were stained with hematoxylin and eosin (Biosharp, Hefei, China) and then scanned using a digital scanner (Konfoong Bioinformation Tech Co. Ltd, Ningbo, CHN) to obtain images.

### 2.8 Measurement of BALF Protein Concentration

The collected BALF was centrifuged, and the supernatant was detected for the protein concentration of BALF using the BCA protein assay kit (Beyotime Biotechnology, Shanghai, China).

### 2.9 Giemsa Staining

Smears of BALF were prepared, and Giemsa staining solution (Beyotime Biotechnology, Shanghai, China) was used for staining, and images were obtained using a digital scanner.

### 2.10 Immunoblotting

The total protein of lung tissues was extracted using RIPA lysis buffer containing protease and phosphatase inhibitors. Protein samples were separated using SDS‐PAGE gels and then transferred onto PVDF membranes (pore size: 0.22 or 0.45 μm, Millipore). The membranes were sealed with a rapid blocking solution and then incubated with primary antibodies at 4°C overnight, followed by incubation with the secondary antibody at room temperature for 1 h. Finally, the protein bands were obtained with an ECL luminescent solution using an Amersham ImageQuant 800 System (Cytiva, China). ImageJ (NIH, Bethesda, MD, USA) was used for grayscale value statistics. β‐actin was used as the loading control for normalization in western blot analysis. The sources and concentrations of antibodies used in this study were provided in Table S1.

### 2.11 Quantitative PCR Analysis

The total mRNA of lung tissues was extracted using Trizol Reagent (Thermo Fisher Scientific, USA). Reverse transcription was performed using PrimeScript RT Master Mix (Takara Biomedical Technology Co. Ltd., Dalian, China). TB Green Premix Ex Taq II (Takara Biomedical Technology Co. Ltd., Dalian, China) was used to obtain amplification values in the CFX384 Touch Real‐Time PCR Detection System (Bio‐Rad Laboratories Inc., USA). Relative mRNA levels of genes were quantified using the 2^−∆∆Ct^ method and normalized to β‐actin (Özturk et al. 2024). For mouse tissue samples, each group contained 6 biological replicates (6 mice per group); for cell samples, each group included 3 biological replicates (3 batches of cells per group). All samples underwent 3 technical replicates on the same culture plate. The primers used in the current study are provided in Table S2.

### 2.12 Immunofluorescence

Lung tissue sections were treated with Triton X‐100, blocked with PBS containing 1% BSA for 1 h, and incubated with primary antibodies at 4°C overnight. A fluorescent secondary antibody was then incubated with slides at room temperature in the dark for 1 h, followed by sealing the slides using a fluorescence quenching sealing agent. Images were obtained using a digital scanner (Konfoong Bioinformation Tech Co. Ltd, Ningbo, CHN).

### 2.13 Network Pharmacology

Combined with the results of UPLC‐MS/MS qualitative analysis of CSF, the targets of the potential active ingredients of CSF were obtained using the Traditional Chinese Medicine Systems Pharmacology Database and Analysis Platform (TCMSP, https://old.tcmsp‐e.com/tcmsp.php) (Ru et al. 2014) and standardized by the UniPort database (http://www.uniprot.org/) (UniProt Consortium 2019). Meanwhile, DrugBank (https://www.drugbank.ca/) (Wishart et al. 2008), GeneCards (https://www.genecards.org/) (Rebhan et al. 1997), and OMIM databases (https://omim.org/) (Amberger et al. 2015) were used to obtain ALI‐relative targets. Subsequently, potential targets of CFS active ingredients for ALI treatment were obtained by overlapping screening. Protein–protein interactions (PPI) analysis of overlapping targets was performed using STRING (version 11.5) data resource (https://cn.string‐db.org/) (Szklarczyk et al. 2017) and visualized by Cytoscape (version 3.10.3). DAVID bioinformatics resource (https://david.ncifcrf.gov/) (Huang et al. 2009) was utilized to further reveal the biological functions of the overlapping targets, including Gene Ontology (GO) enrichment and Kyoto Encyclopedia of Genes and Genomes (KEGG) pathway analysis.

### 2.14 Molecular Docking

PubChem database (https://pubchem.ncbi.nlm.nih.gov/, accessed on October 30, 2024) (Wang et al. 2009) was used to obtain the structures of CSF active ingredients. Meanwhile, the 3D structure of NF‐κB p65 (PDB_ID: 1NFI) and NLRP3 (PDB_ID: 6NPY) were obtained from the RCSB PDB database (https://www.rcsb.org/, accessed on October 30, 2024) (Berman et al. 2000). These structures were processed using PyMOL software 3.1.3.1, including dehydrogenation, dephosphorylation, and removal of symmetrical chains. Subsequently, the target proteins were imported into Auto Dock Tools 1.5.7 for hydrogenation, charge assignment, and atom type assignment. Molecular docking analysis was performed using Auto Dock Vina 1.2.3 (Trott and Olson 2010) and PyMOL was used to visualize the results. For NF‐κB p65 (1NFI), the grid box was centered at coordinates (*x* = −5.070, *y* = 69.905, *z* = 50.028) with dimensions *x* = 70, *y* = 82, *z* = 126; for NLRP3 (6NPY), the grid box was centered at coordinates (*x* = 107.580, *y* = 98.357, *z* = 109.629) with dimensions *x* = 126, *y* = 112, *z* = 126.

### 2.15 Molecular Dynamics Simulation

To further investigate the molecular mechanism of binding of CSF active ingredients to ALI target proteins, molecular dynamics simulations of the complexes of Bergapten, Hesperetin, Hesperidin, and Naringin with NF‐κB p65 and NLRP3 were performed using the Gromacs 2020 software package. As previously described (Luo et al. 2022), the protein was assigned AMBER99SB‐ILDN force field parameters, while small molecule ligands were assigned General Amber Force Field (GAFF) parameters. The small molecule topologies were constructed using the Sobtop program, with Restrained Electrostatic Potential (RESP) charge fitting performed. The TIP3P explicit water model was selected, with a minimum distance of 1.0 nm between atoms of the protein and the edges of the water box. Based on the docking results, sodium or chloride ions were used to neutralize the system charge. Protein (and compound) heavy atoms were constrained, and energy minimization was performed on the water molecules. Subsequently, the constraint was released, and energy minimization was conducted on the whole system. The system was heated slowly to 300 K and equilibrated at NPT. Finally, a 100 ns molecular dynamics (MD) simulation of the system was performed, with trajectory data saved every 10 ps and correlation analysis performed using the trjconv module. The gmx_MMPBSA method of the Gromacs 2020 program was used to calculate the binding free energies of ligands and proteins.

### 2.16 Statistical Analysis

Data were visualized using GraphPad Prism 9.0 software (San Diego, CA, USA). Statistical analyses were performed using IBM SPSS Statistics 26 software. The normality of data distribution was first evaluated by the Shapiro–Wilk test. For data conforming to normal distribution, homogeneity of variance was further tested using Levene's test. If the variance was homogeneous, one‐way analysis of variance (One‐way ANOVA) was conducted first, followed by the least significant difference (LSD) test for pairwise comparisons. If the variance was heterogeneous, one‐way ANOVA was performed, and then Dunnett's post hoc test was used for comparisons. For data that did not conform to normal distribution, non‐parametric tests (Kruskal–Wallis H test for multiple group comparisons, with Dunn's test for subsequent pairwise comparisons when appropriate) were applied. Results were presented as mean ± SEM. *p* < 0.05 was considered statistically different.

## 3 Results

### 3.1 Identification of CSF Active Ingredients

**FIGURE 1:**
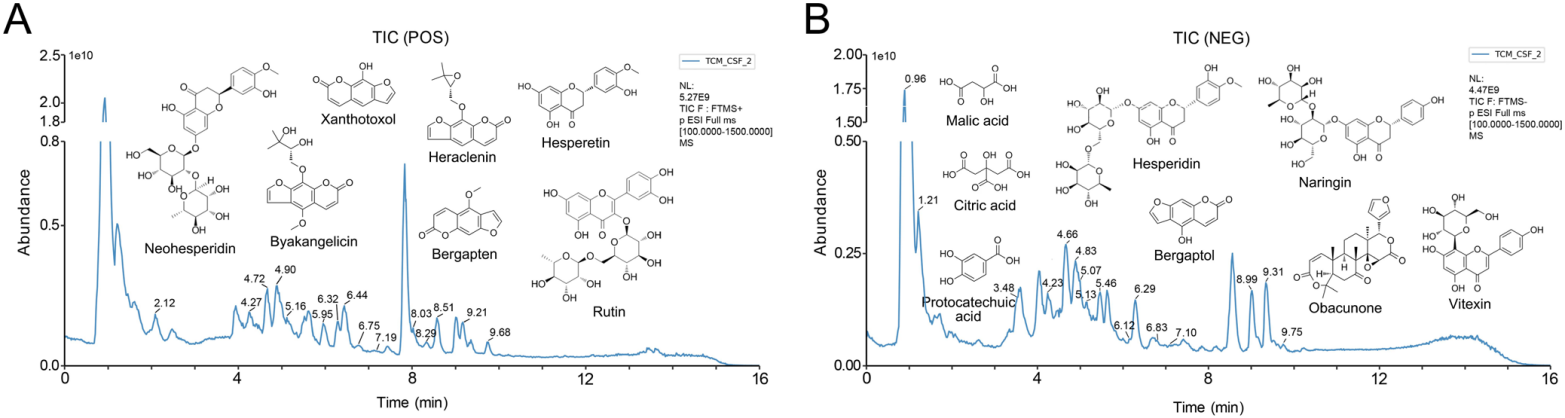
Overview of 31 compounds of ECG identified by UPLC‐MS/MS analysis. (A) TIC of CSF: Positive ion mode. Compounds were sequentially identified as: 3,5‐dihydroxy‐6‐methyl‐2,3‐dihydropyran‐4‐one, 5‐Hydroxyferulic acid, rutin, 2‐Methyl‐5‐carboxymethyl‐7‐hydroxychromone, neohesperidin, xanthotoxol, syringaresinol, byakangelicin, herniarin, hesperetin, bergapten, heraclenin, limonin, 15,16‐bisnor‐13‐oxo‐8(17)‐labden‐19‐oicacid, and tangeretin. (B) TIC of CSF: Negative ion mode. Compounds were sequentially identified as: malic acid, citric acid, protocatechuic acid, scopolin, sweroside, vitexin, naringin, hesperidin, 2‐hydroxycinnamic acid, luteolin, bergaptol, naringenin, diosmetin, 4‐methoxy‐5‐hydroxybisabola‐2,10‐diene‐9‐one, demethylsuberosin, and obacunone.

To clarify the material basis for the functional effects of CSF, the CSF samples were analyzed using UPLC‐MS/MS combined with the LutMet‐CM1.0 database and herb database by Shanghai OE Biotech Co. Ltd. (Shanghai, China). In the present study, 31 main active ingredients of CSF were identified (Table S3). As shown in Figure 1, 3,5‐dihydroxy‐6‐methyl‐2,3‐dihydropyran‐4‐one, 5‐Hydroxyferulic acid, rutin, 2‐Methyl‐5‐carboxymethyl‐7‐hydroxychromone, neohesperidin, xanthotoxol, syringaresinol, byakangelicin, herniarin, hesperetin, bergapten, heraclenin, limonin, 15,16‐bisnor‐13‐oxo‐8(17)‐labden‐19‐oic acid, and tangeretin were identified in the positive ion mode, as well as malic acid, citric acid, protocatechuic acid, scopolin, sweroside, vitexin, naringin, hesperidin, 2‐hydroxycinnamic acid, luteolin, bergaptol, naringenin, diosmetin, 4‐methoxy‐5‐hydroxybisabola‐2,10‐diene‐9‐one, demethylsuberosin, and obacunone in the negative ion mode. These findings help to reveal the functional mechanisms of CSF to ameliorate ALI.

### 3.2 CSF Presented a Protective Effect Against LPS‐Induced ALI in Mice

**FIGURE 2:**
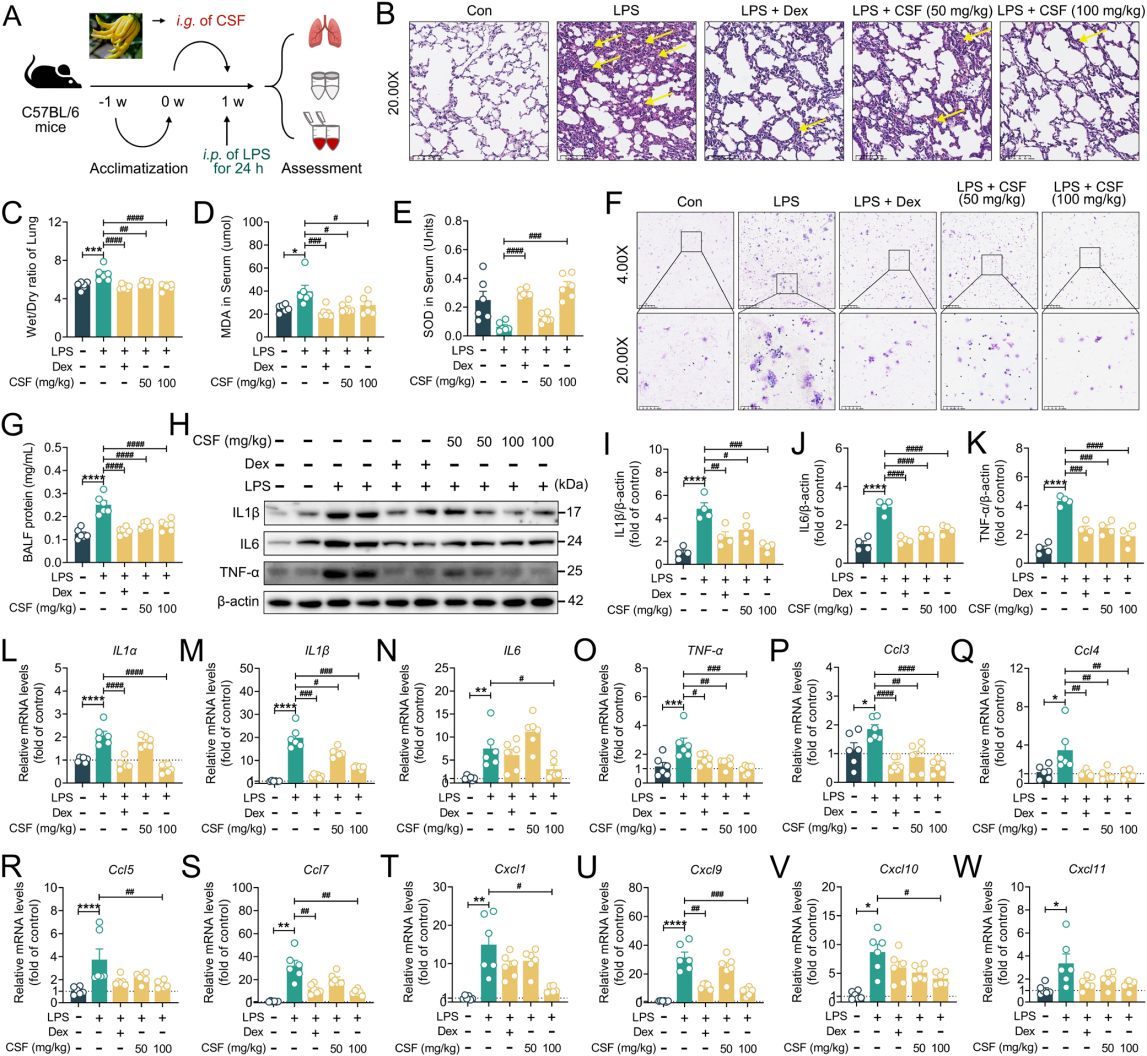
CSF presented a protective effect against LPS‐induced ALI in mice. (A) Experimental process. (B) Representative images of H&E staining in lung tissues. Original magnification, 20.00×. (C) Wet/Dry radio of lung (*n* = 6). (D, E) MDA and SOD levels in serum (*n* = 6). (F) Giemsa staining in BALF. Original magnification, 4.00× and 20.00×. (G) BALF protein level (*n* = 6). (H–K) Representative immunoblotting and quantification of IL1β, IL6, and TNF‐α in lung tissues (*n* = 4). (L–O) mRNA level of pro‐inflammatory cytokines *IL1α*, *IL1β*, *IL6*, and *TNF‐α* in lung tissues (*n* = 6). (P–W) mRNA level of chemokines *Ccl3*, *Ccl4*, *Ccl5*, *Ccl7*, *Cxcl1*, *Cxcl9*, *Cxcl10*, and *Cxcl10* in lung tissues (*n* = 6). Data are presented as mean ± SEM. *p < 0.05, **p < 0.01***, *p* < 0.001, and ****p < 0.0001 versus the control group. ^#^ *p* < 0.05, ^##^ *p* < 0.01, ^###^ *p* < 0.001, and ^####^ *p* < 0.0001 versus the LPS group.

Previous studies have demonstrated that CSF exerts strong anti‐inflammatory effects (Kim et al. 2013). In the current study, mice were orally administered (i.g.) with CSF for 1 week, followed by intraperitoneal injection of LPS, and samples were collected 24 h later to investigate whether CSF could ameliorate LPS‐induced ALI (Figure 2A). As shown in Figure 2B,C, compared with normal control mice, LPS‐challenged mice exhibited pronounced neutrophil infiltration, inflammatory cell accumulation, and pulmonary edema in lung tissue. In contrast, CSF pretreatment significantly attenuated these pathological changes (Figure S1). MDA is the product of lipid peroxidation closely related to the pathogenesis of ALI. Notably, CSF markedly reduced serum MDA levels and simultaneously increased SOD levels in ALI mice (Figure 2D,E). In addition, CSF significantly reduced macrophage and neutrophil counts, as well as protein levels in BALF, and attenuated lung tissue protein leakage to a similar extent as Dex pretreatment (Figure 2F,G). Further studies revealed that CSF not only effectively suppressed lung tissue expression of IL1β, IL6, and TNF‐α proteins (Figure 2H–K), but also reduced *IL1α*, *IL1β*, *IL6*, and *TNF‐α* mRNA levels (Figure 2L–O). Meanwhile, CSF also excellently reduced lung tissue chemokines *Ccl3*, *Ccl4*, *Ccl5*, *Ccl7*, *Cxcl1*, *Cxcl9*, *Cxcl10*, and *Cxcl11* mRNA levels (Figure 2P–W), exhibiting anti‐inflammatory functional effects comparable to those of Dex. These results illustrate that CSF presents a remarkable protective effect against LPS‐induced ALI in mice.

### 3.3 CSF Ameliorated LPS‐Stimulated ALI in Mice by Inhibiting NF‐κB Signaling

**FIGURE 3:**
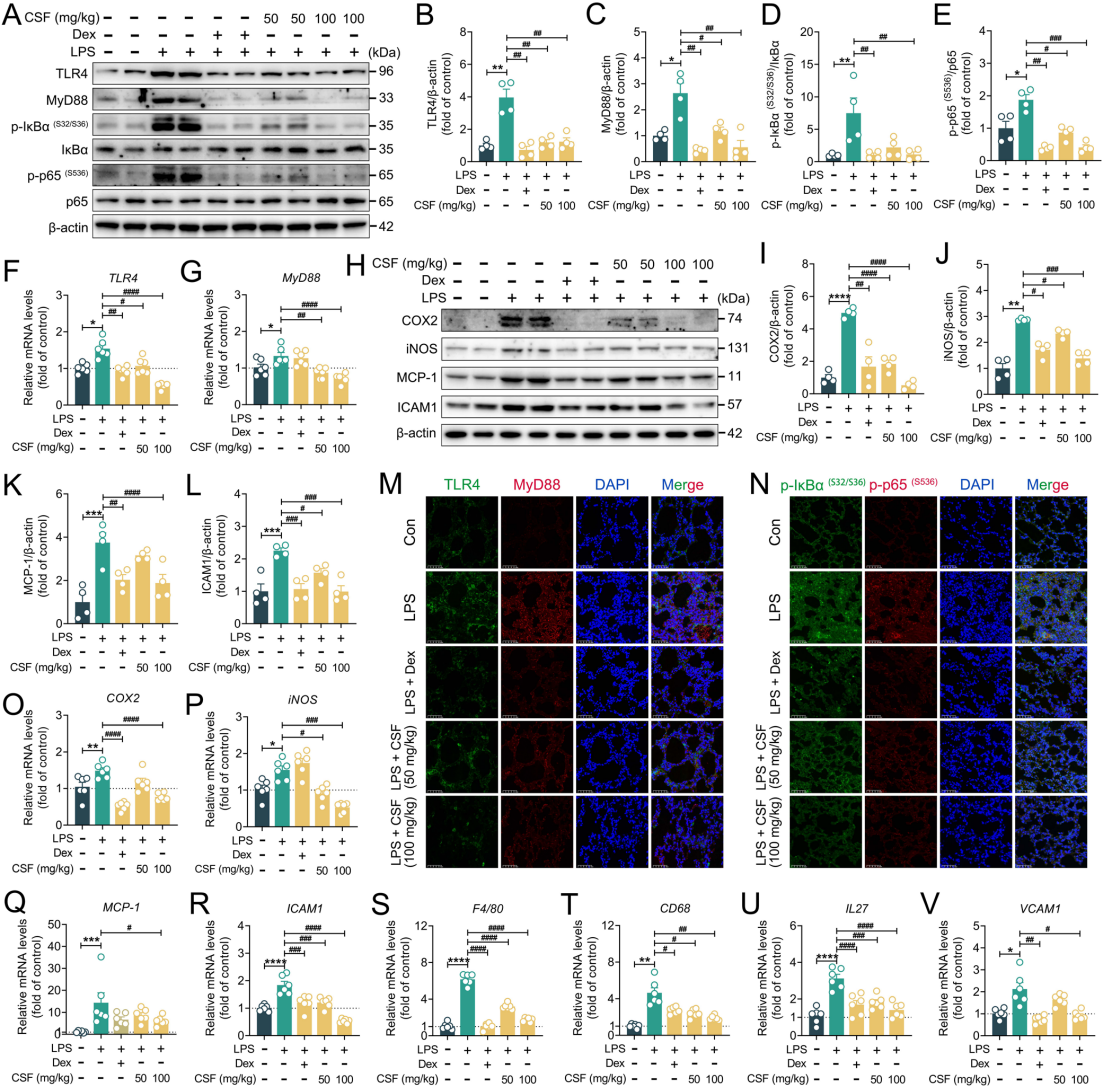
CSF ameliorated LPS‐stimulated ALI in mice by inhibiting NF‐κB signaling. (A–E) Representative immunoblotting and quantification of TLR4, MyD88, p‐IκBα (S32/S36), IκBα, p‐p65 (S536), and p65 in lung tissues (*n* = 4). (F, G) mRNA levels of *TLR4* and *MyD88* in lung tissues (*n* = 6). (H–L) Representative immunoblotting and quantification of COX2, iNOS, MCP‐1, and ICAM1 in lung tissues (*n* = 4). (M, N) Representative immunofluorescence images of TLR4‐positive, MyD88‐positive, p‐IκBα (S32/S36)‐positive, and p‐p65 (S536)‐positive in lung tissues. Scale bars: 50 μm. (O–V) mRNA levels of *COX2*, *iNOS*, *MCP‐1*, *ICAM1*, *F4/80*, *CD68*, *IL27*, and *VCAM1* in lung tissues (*n* = 6). Data are presented as mean ± SEM. *p < 0.05, **p < 0.01, ***p < 0.001, and ****p < 0.0001 versus the control group. ^#^ *p* < 0.05, ^##^ *p* < 0.01, ^###^ *p* < 0.001, and ^####^ *p* < 0.0001 versus the LPS group.

Given that CSF effectively reduced the production of pro‐inflammatory cytokines and chemokines in lung tissues of mice with LPS‐induced ALI, and that LPS‐stimulated classical NF‐κB signaling is a key driver of ALI pathogenesis and progression (Quinton and Mizgerd 2011; Quinton et al. 2018), this study further investigated whether CSF could block the TLR4/MyD88/NF‐κB p65 signaling pathway using immunoblotting, immunofluorescence, and qPCR. As shown in Figure 3A–E, LPS‐stimulated upregulation of TLR4, MyD88, p‐IκBα (S32/S36), and p‐p65 (S536) proteins was markedly inhibited by Dex and CSF intervention, while consistent results were observed at the gene levels of *TLR4* and *MyD88* (Figure 3F,G). Additionally, analysis of downstream signaling revealed that CSF also markedly reduced the expression of COX2, iNOS, ICAM1, and MCP‐1 proteins (Figure 3H–L). Further immunofluorescence and qPCR results also confirmed the capacity of CSF to suppress LPS‐stimulated NF‐κB classical signaling. CSF significantly inhibited the fluorescent expression of TLR4, MyD88, p‐IκBα (S32/S36), and p‐p65 (S536) in lung tissues of mice with ALI (Figure 3M,N), as well as decreased the gene levels of inflammatory mediators, such as *COX2*, *iNOS*, *MCP‐1*, *ICAM1*, *F4/80*, *CD68*, *IL27*, and *VCAM1* (Figure 3O–V). These findings indicate that CSF attenuates LPS‐stimulated ALI in mice by inhibiting classical NF‐κB inflammatory signaling, demonstrating its potent anti‐inflammatory ability.

### 3.4 CSF Attenuated LPS‐Induced ALI in Mice by Suppressing NLRP3 Inflammasome Activation

**FIGURE 4:**
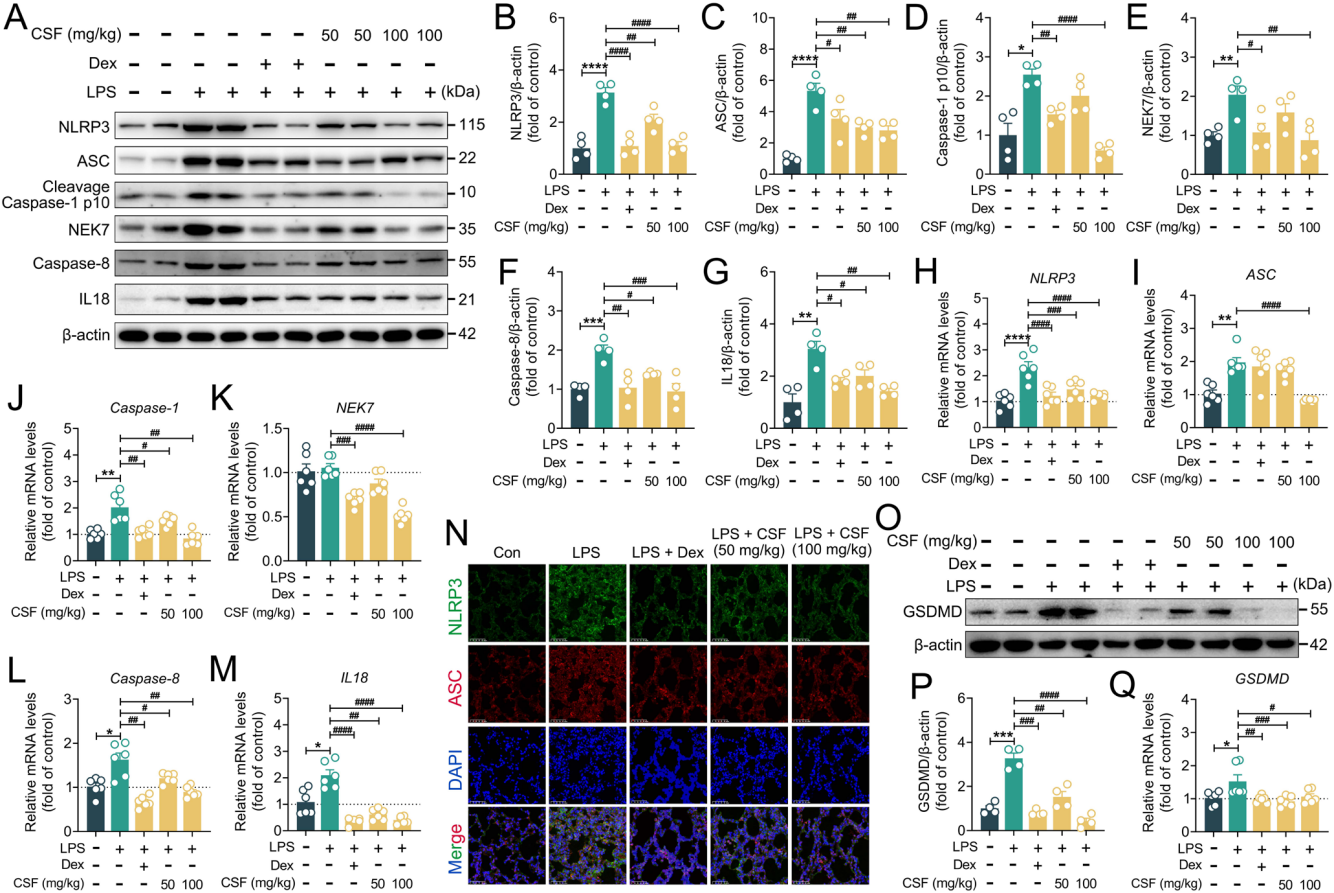
CSF attenuated LPS‐induced ALI in mice by suppressing NLRP3 inflammasome activation. (A–G) Representative immunoblotting and quantification of NLRP3, ASC, cleavage Caspase‐1 p10, NEK7, Caspase‐8, and IL18 in lung tissues (*n* = 4). (H–M) mRNA levels of *NLRP3*, *ASC*, *Caspase‐1*, *NEK7*, *Caspase‐8*, and *IL18* in lung tissues (*n* = 6). (N) Representative immunofluorescence images of NLRP3‐positive, ASC‐positive in lung tissues. Scale bars: 50 μm. (O, P) Representative immunoblotting and quantification of GSDMD in lung tissues (*n* = 4). (Q) mRNA levels of *GSDMD* in lung tissues (*n* = 6). Data are presented as mean ± SEM. *p < 0.05, **p < 0.01, ***p < 0.001, and ****p < 0.0001 versus the control group. ^#^ *p* < 0.05, ^##^ *p* < 0.01, ^###^ *p* < 0.001, and ^####^ *p* < 0.0001 versus the LPS group.

As a key mediator of the inflammatory response, the LPS‐activated transcription factor NF‐κB modulates innate and adaptive immune functions (Liu et al. 2017). NF‐κB signal transduction is involved in activating the NLRP3 inflammasome, thereby accelerating the onset and progression of inflammatory diseases (Guo et al. 2015). To further explore the protective mechanisms of CSF intervention against LPS‐induced ALI in mice, the expression levels of key proteins and genes for NLRP3 inflammasome activation were analyzed by immunoblotting, qPCR, and immunofluorescence. As shown in Figure 4A–M, CSF significantly downregulated the protein and mRNA levels of NLRP3, ASC, Caspase‐1 p10, NEK7, Caspase‐8, and IL18, with consistent results confirmed by immunofluorescence (Figure 4N). Notably, the above results have demonstrated the ability of CSF to inhibit the secretion of mature IL1β (Figure 2H,K,M). Besides, CSF markedly reduced GSDMD gene mRNA and protein levels (Figure 4O–Q), indicating that it could also ameliorate ALI by mediating cellular pyroptosis. These findings suggest that CSF alleviates LPS‐induced ALI in mice by inhibiting the activation of the classical inflammasome.

### 3.5 Network Pharmacology Analysis of CSF to Ameliorate ALI

**FIGURE 5:**
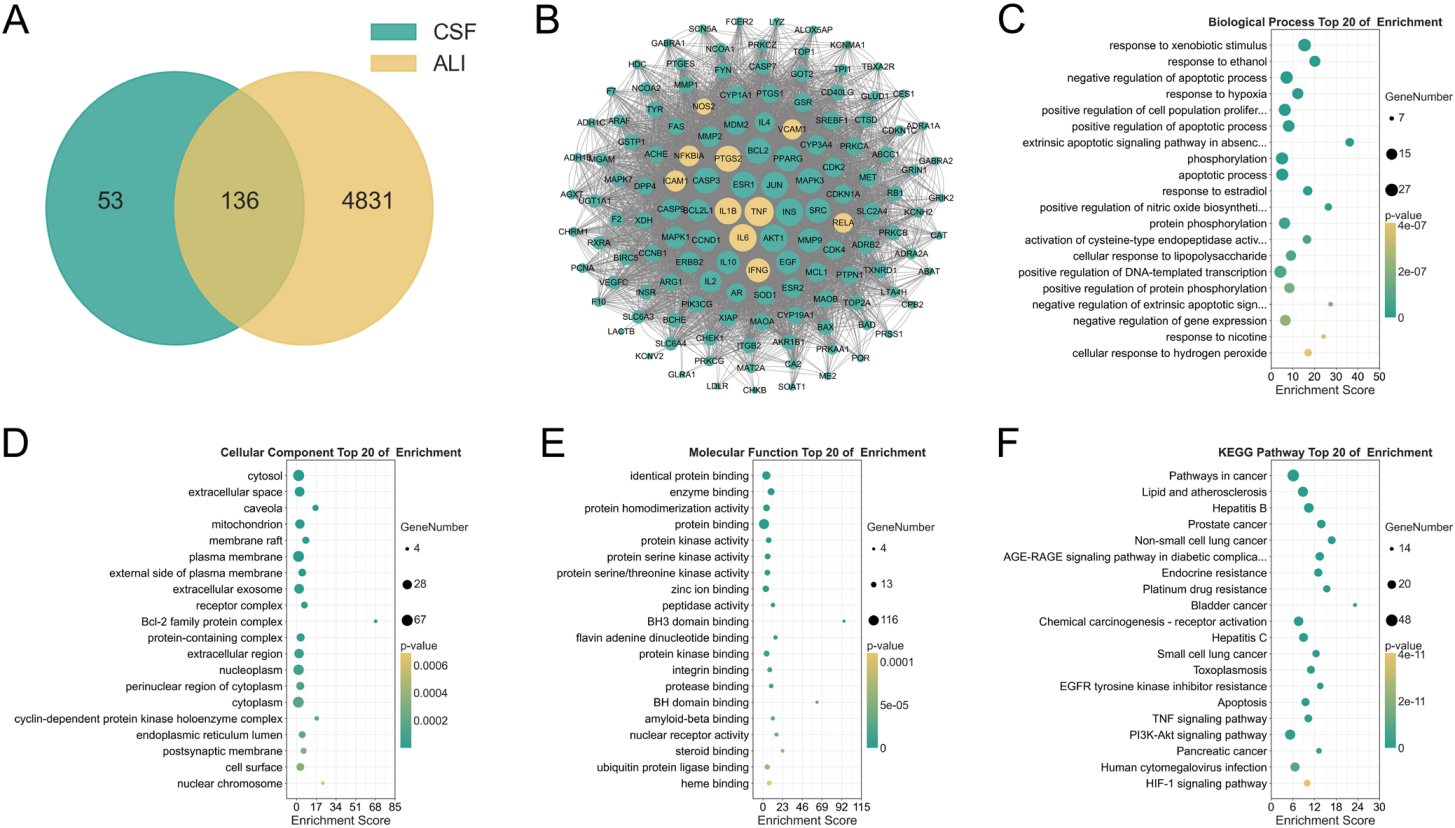
Network pharmacology analysis of CSF to ameliorate ALI. (A) Venn diagram of CSF and ALI‐related targets. (B) PPI network of potential targets. (C) Biological processes. (D) Cellular components. (E) Molecular function. (F) KEGG pathway.

Given that CSF alleviates LPS‐induced ALI in mice through multi‐component, multi‐target, and multi‐pathway mechanisms, network pharmacology analysis was performed to systematically illustrate the interactions among active ingredients, targets, and pathways, aiming to present these connections more comprehensively and intuitively. As shown in Figure 5A, 136 overlapping targets were screened by intersecting CSF active ingredient targets with ALI‐associated targets. Further PPI analysis of these targets revealed that 6052 edges were presented, of which TNF (degree = 218), IL6 (degree = 202), IL1β (degree = 194), PTGS2 (degree = 188), INFG (degree = 168), NFKBIA (degree = 140), ICAM1 (degree = 140), VCAM1 (degree = 132), RELA (degree = 128), and NOS2 (degree = 96) inflammatory targets had higher degree values (Figure 5B), suggesting that these targets were co‐involved in the onset and progression of ALI. In addition, the results of GO enrichment analysis indicated that the targets of CSF active ingredients to ameliorate ALI were mainly associated with cellular response to lipopolysaccharide, negative regulation of gene expression, and negative regulation of apoptotic process in biological process, cytoplasm, mitochondrion, and protein‐containing complex in cellular component, and enzyme binding, protein binding, and protein kinase binding in molecular function. Further KEGG pathway analysis revealed that these targets mainly participated in the TNF signaling pathway, apoptosis, and the HIF‐1 signaling pathway. Indeed, the TNF signaling contains key target proteins of the NF‐κB signaling pathway, such as RELA, NFKBIA, etc. (Figure 5C–F). Taken together, network functional analysis suggests that CSF active ingredients exert an essential effect in modulating inflammatory signaling activation and transduction.

### 3.6 CSF Active Ingredient Exerted Stable Binding to NF‐κB p65 and NLRP3 to Ameliorate ALI

**FIGURE 6:**
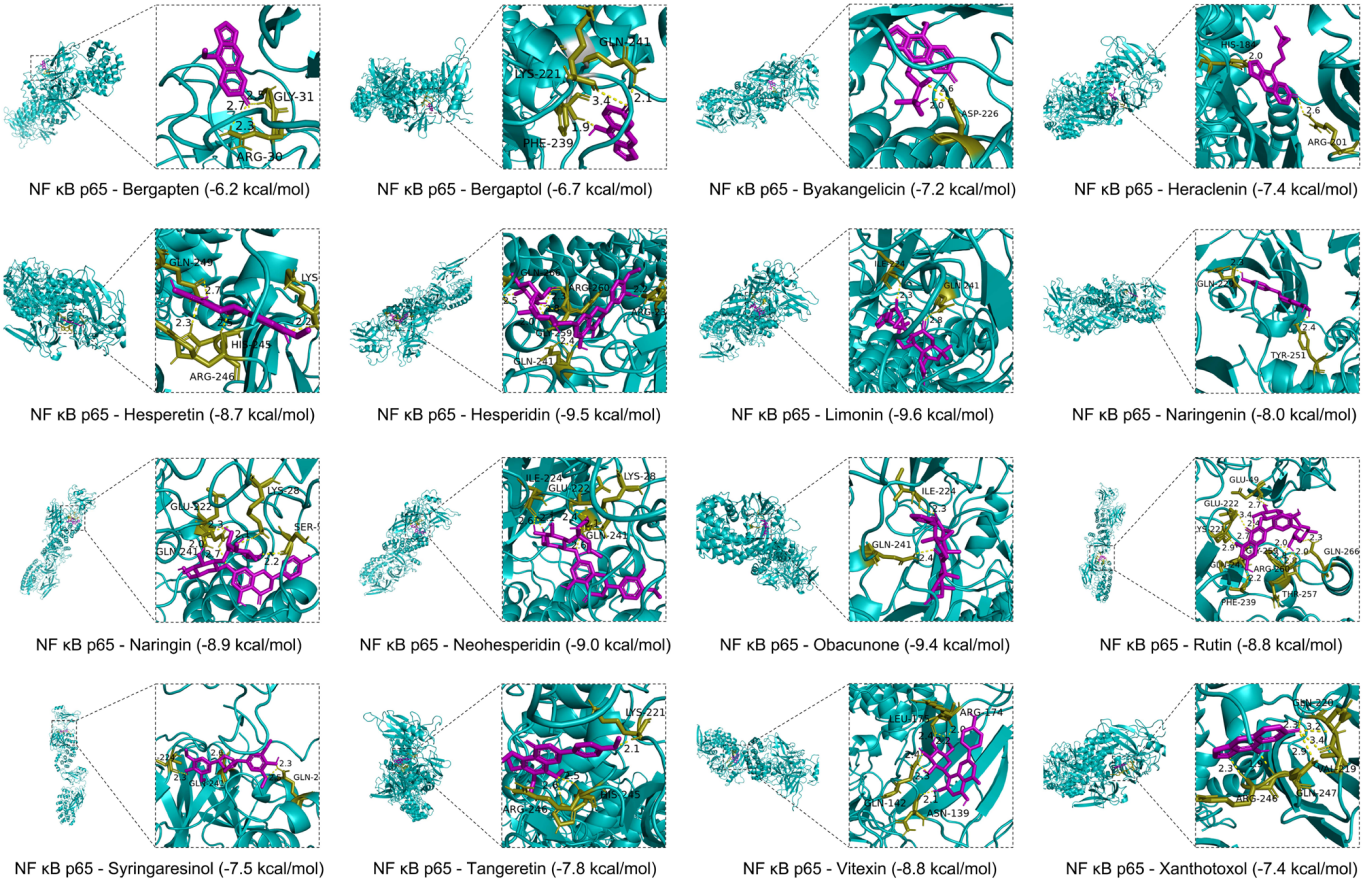
Molecular docking analysis of CSF active ingredients with NF‐κB p65 (PDB_ID: 1NFI).

**FIGURE 7:**
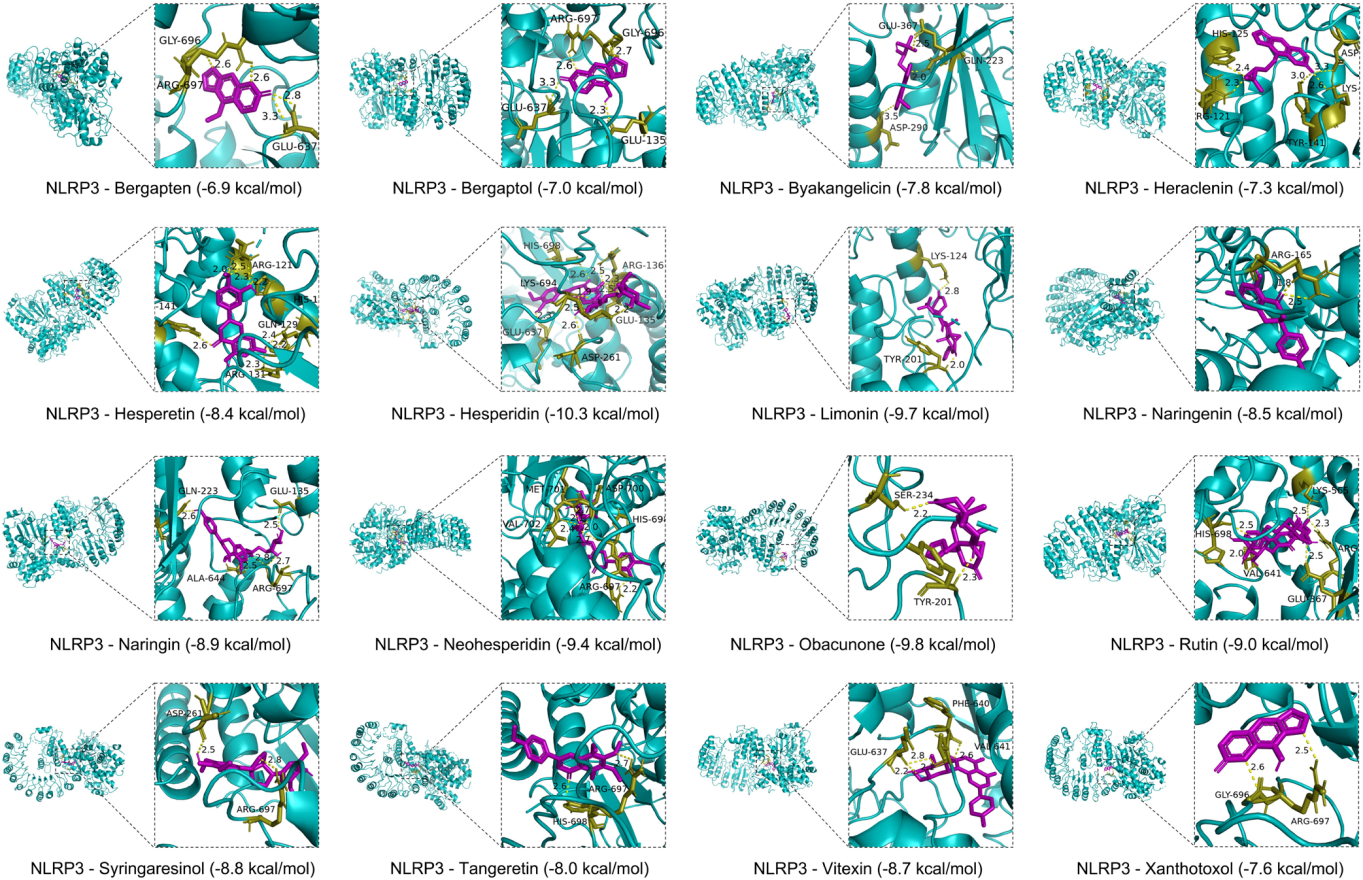
Molecular docking analysis of CSF active ingredients with NLRP3 (PDB_ID: 6NPY).

Given the critical role of NF‐κB p65 and NLRP3 inflammasome in ALI pathogenesis (Gross et al. 2018; Grailer et al. 2014), we examined whether the key active ingredients identified in CSF samples bind stably to NF‐κB p65 or NLRP3 and thus exert their beneficial inhibitory effects and therapeutic implications. As shown in Figures 6 and 7, the docking binding energies of the 16 selected active ingredients with NF‐κB p65 and NLRP3 were all less than −5 kcal/mol, ranging from −10.3 to −6.2 kcal/mol. More detailed information, including 2D images, is presented in Table S4. These results indicate that these active ingredients of CSF have high binding affinities with NF‐κB p65 and NLRP3, which may inhibit the activation of inflammation signaling pathways and alleviate the development and progression of ALI.

### 3.7 MD Simulations Confirmed the Stability of CSF Active Ingredients Binding to NF‐κB p65 and NLRP3

**FIGURE 8:**
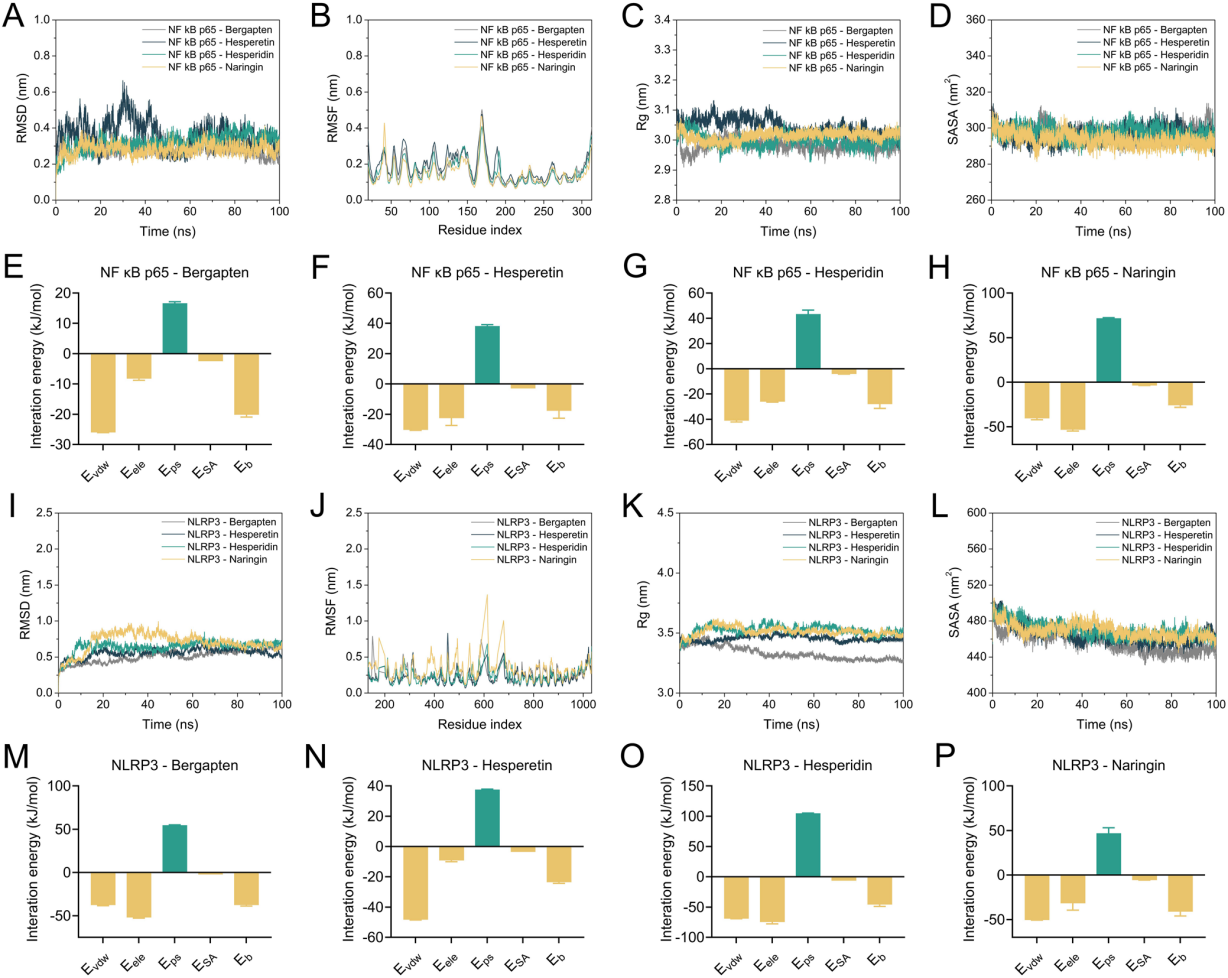
Profiles of molecular dynamics simulations. (A) RMSD analysis for the NF‐κB p65—ligand complexes. (B) RMSF analysis for the NF‐κB p65—ligand complexes. (C) The plot of Rg of NF‐κB p65—ligand complexes varies with time. (D) The plot of SASA of NF‐κB p65—ligand complexes. (E–H) Binding free energy decomposition of NF‐κB p65—Bergapten, NF‐κB p65—Hesperetin, NF‐κB p65—Hesperidin, and NF‐κB p65—Naringin complex. (I) RMSD analysis for the NLRP3—ligand complexes. (J) RMSF analysis for the NLRP3—ligand complexes. (K) The plot of Rg of NLRP3—ligand complexes varies with time. (L) The plot of SASA of NLRP3—ligand complexes. (M–P) Binding free energy decomposition of NLRP3—Bergapten, NLRP3—Hesperetin, NLRP3—Hesperidin, and NLRP3—Naringin complex.

The above molecular docking results comprehensively demonstrate the binding modes of CSF active ingredients to the critical proteins involved in ALI progression, characterized by low binding free energies. In the current study, further MD simulations confirmed its stability in the binding pocket. As shown in the root mean square deviation (RMSD) analysis, all systems were stabilized within 100 ns; only the NF‐κB p65—Hesperetin system exhibited a slight fluctuation at 30 ns (Figure 8A). Notably, the root mean square fluctuation (RMSF), the radius of gyration (Rg), and solvent‐accessible surface area (SASA) analysis showed that the NF‐κB p65 binding to Bergapten, Hesperetin, Hesperidin, and Naringin systems was stable within 100 ns of MD simulations and exhibited lower binding free energies (Figure 8B–H), including van der Waals energy (E_vd_), electrostatic energy (E_ele_), polar solvation energy (E_ps_), SASA energy (E_SA_) and binding energy (E_b_) (Table S5). Interestingly, similar results were observed in MD simulations of complex systems involving NLRP3 with Bergapten, Hesperetin, Hesperidin, and Naringin (Figure 8I–P). These findings suggest that CSF active ingredients exert a significant inhibitory effect on inflammatory signaling by affecting the function of key proteins NF‐κB p65 and NLRP3 involved in ALI pathogenesis, thereby attenuating the progression of ALI.

## 4 Discussion

ALI/acute respiratory distress syndrome (ARDS) is a common life‐threatening respiratory disease, clinically manifested by progressive hypoxemia and respiratory distress, with extremely high morbidity and mortality (Meyer et al. 2021; Bellani et al. 2016). Previous studies have long shown that ALI/ARDS can be triggered by a variety of risk factors, while severe lung infection is a direct contributor to lung injury. LPS, a bacterial endotoxin with the function of inducing acute inflammation, has been widely used to induce ALI in mice by intranasal administration or intraperitoneal injection (Qian et al. 2022; Xu et al. 2024). The present study aims to investigate the functional efficacy and implication of CSF in LPS‐induced ALI and systematically reveal the underlying mechanisms.

CSF, an ornamental and edible fruit from *Citrus medica* L. var. *sarcodactylis Swingle* of the Rutaceae family, has a long history of medicinal application and is rich in flavonoids, coumarins, organic acids, and limonoid compounds with excellent anti‐inflammatory and antioxidant functional properties (Wang et al. 2021). Consistent with this, the CSF used in our study was found to contain abundant flavonoids (including neohesperidin, hesperetin, tangeretin, among others), coumarins (including xanthotoxol, byakangelicin, bergapten, among others), and organic acids (including malic acid, 5‐Hydroxyferulic acid, citric acid, among others). Most of them have been reported to exert ameliorative effects on pulmonary inflammation through multiple pathways, suggesting that CSF containing these active ingredients may also have ameliorative effects on ALI (Dong et al. 2020; Liao et al. 2024; Guo et al. 2019).

Dex, with its potent and well‐defined anti‐inflammatory effects, is a commonly used positive control drug in ALI studies, and it was also selected as the positive control in our research (Zhao et al. 2025; Li et al. 2023). The LPS‐induced ALI model is similarly widely used, with numerous studies employing this model to investigate drugs' ameliorative effects on ALI (Xu et al. 2025; Yang et al. 2020). As expected, LPS successfully induced the pathological features of ALI, including increased permeability of the pulmonary capillary endothelial and alveolar epithelial barriers, which led to edema in alveoli and interstitial spaces, along with promoted protein leakage and infiltration of various inflammatory cells (predominantly neutrophils). In contrast, DEX effectively inhibited multiple inflammatory mediators, including proinflammatory cytokines, chemokines, peroxidases, and proteases, while exerting its effects by suppressing macrophage/neutrophil‐mediated lung injury. Interestingly, we are the first to observe that CSF also exhibits functional efficacy comparable to that of Dex.

Acute inflammation, an important process in the progression of lung injury, is closely linked to the activation of classical NF‐κB signaling (Liu et al. 2017). LPS is recognized by lipopolysaccharide‐binding proteins (LBP) and binds to CD14 to form the LPS‐LBP‐CD14 triplex, activates TLR4 of immune cells, thereby triggering NF‐κB signaling. Upon activation of NF‐κB dimers (mainly p50/p65), IκB is phosphorylated and degraded, allowing NF‐κB dimers to translocate into the nucleus and regulate the expression of various inflammatory mediators (Chow et al. 1999). In exploring how CSF attenuates LPS‐induced ALI in mice, we found it significantly inhibited LPS‐stimulated TLR4/MyD88/NF‐κB p65 signaling, reduced IκBα phosphorylation and degradation, and prevented NF‐κB p65 nuclear translocation. In fact, inhibiting NF‐κB acts as a therapeutic focus for plant‐derived nutraceuticals in alleviating inflammatory lung conditions (Alharbi et al. 2022). Correspondingly, the subsequent finding that CSF inhibits the transcription and expression of inflammatory mediators further confirms the role of NF‐κB activity inhibition in ameliorating pulmonary inflammation. These results partially revealed the molecular mechanisms of CSF to ameliorate ALI and also confirmed its excellent ability to resist the onset and progression of acute lung inflammation.

Inflammasome, a multiprotein polymeric complex, plays a critical role in the innate immune response induced by pathogen invasion or tissue damage (Zhang et al. 2023). Previous studies have long demonstrated that NLRP3 inflammasome activation is closely associated with ALI (Grailer et al. 2014). Activation of the NLRP3 inflammasome requires both priming and activation signals. Specifically, LPS‐stimulated TLR4/NF‐κB signaling provides priming signals, which promote the assembly and activation of the NLRP3 inflammasome, as well as the transcription and translation of cytokines (Zhang et al. 2023). Previous studies have demonstrated the potential to attenuate LPS‐induced mouse ALI by inhibiting the NLRP3/ASC/caspase‐1 axis (Wang et al. 2025). Similarly, the results of this study unequivocally revealed that CSF markedly inhibited the assembly of NLRP3/ASC/Caspase‐1 zymogens into inflammasome and suppressed Caspase‐1 activation, thereby reducing maturation and release of the inflammatory cytokines IL1β and IL18. Also, proteolytic cleavage of GSDMD by inflammatory caspases is the key factor that governs pyroptotic cell death (Shi et al. 2015). As expected, CSF regulated Caspase‐1‐mediated cleavage of GSDMD and inhibited pyroptosis. These findings further reveal the molecular mechanisms of CSF attenuating LPS‐induced ALI and re‐emphasize the strong anti‐inflammatory functional effects of CSF.

In the current study, 31 components of CSF were qualitatively identified. Meanwhile, combining the TCMSP data platform and Drugbank, Genecards, and OMIM disease target databases, further network functional analysis revealed that the active ingredient of CSF could regulate targets closely related to inflammation, including TNF, IL1β, IL6, RELA, NFKBIA, INFG, ICAM1, VCAM1, etc. These targets, identified via network functional analysis, are primarily involved in pathways including the TNF signaling pathway, apoptosis, and the HIF‐1 signaling pathway. The TNF signaling pathway (linked to the NF‐κB pathway) is a key driver of inflammatory cascades in ALI, promoting the release of proinflammatory factors and infiltration of inflammatory cells, thereby exacerbating lung injury (Tang et al. 2021). Aberrant activation of the apoptosis pathway induces apoptosis of alveolar epithelial and pulmonary vascular endothelial cells, impairing lung barrier integrity and triggering pathological changes such as pulmonary edema (Ge et al. 2024). The HIF‐1 signaling pathway is activated under hypoxic conditions in ALI, and its excessive activation exacerbates pulmonary inflammation and edema by upregulating proinflammatory factors, among other mechanisms (Dunham‐Snary et al. 2017). These pathways each regulate inflammatory amplification, cell apoptosis, and hypoxic adaptation, collectively contributing to the process of lung injury in ALI. These findings were consistent with the results of animal experiments and confirmed the multi‐component, multi‐target, multi‐pathway functional mechanisms of CSF in ameliorating ALI.

Previous studies have shown that molecular docking and molecular dynamics simulation techniques play a significant role in drug development, as they provide in‐depth elaboration of intermolecular interactions and graphical illustration of interaction mechanisms (Alonso et al. 2006; Adamu et al. 2022). Thus, further studies on the active ingredients and targets can help reveal more systematically the functional mechanisms of CSF in ameliorating ALI. Interestingly, the current study found that all 16 active ingredients of CSF bind to the key targets of ALI occurrence and progression, NF‐κB p65 and NLRP3, with a free energy of less than −5 kcal/mol. Moreover, MD simulations demonstrated that the key active ingredients of CSF, Bergapten, Hesperetin, Hesperidin, and Naringin were stable in the binding pockets and showed lower binding free energies with NF‐κB p65 and NLRP3, respectively. These findings further suggest that CSF exerts the functional properties of multiple active ingredients and multiple targets to alleviate ALI.

The present study has certain limitations in elucidating CSF‐specific functional mechanisms specifically, including the lack of pharmacokinetic data, absence of isolation of individual compounds, scarcity of cell line‐based mechanistic assays, and insufficient long‐term safety data. Therefore, further in‐depth studies are needed to reveal the molecular mechanisms of CSF to ameliorate ALI.

## 5 Conclusion

**FIGURE 9:**
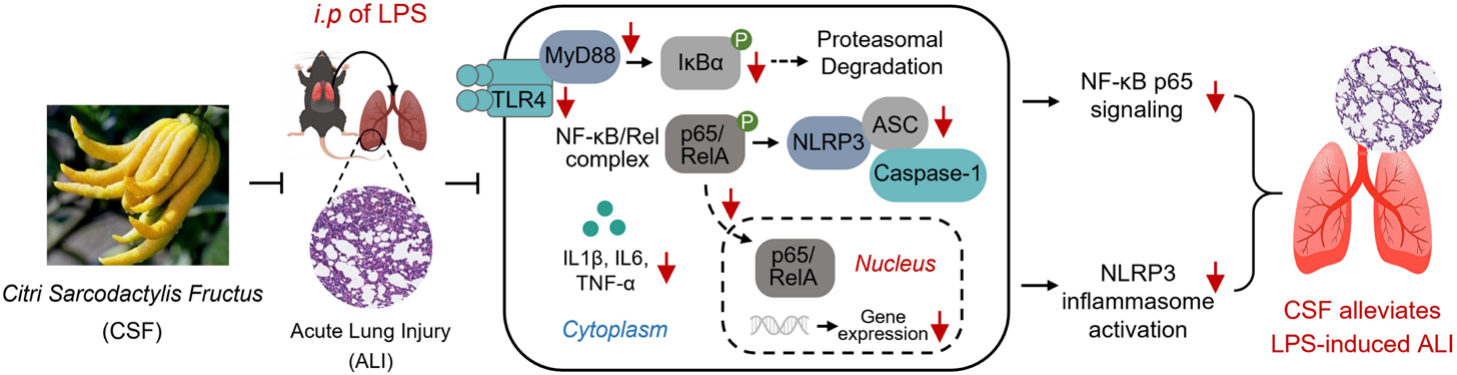
The molecular mechanism diagram of CSF alleviated LPS‐induced ALI in this study.

In summary, the present findings have provided strong evidence that CSF alleviates LPS‐induced ALI by inhibiting NF‐κB signaling and the NLRP3 inflammasome activation‐mediated inflammatory response (Figure 9). It also emphasized the characteristics of traditional Chinese medicine whereby CSF exerts its functional effects through multi‐components, multi‐targets, and multi‐pathways. These results provide a scientific basis for the utilization of CSF resources.

## Author Contributions

**Zhuohui Luo:** conceptualization (equal), investigation (equal), methodology (equal), visualization (equal), writing – review and editing (equal). **Zaibin Xu:** investigation (equal), writing – review and editing (equal). **Kongyan Wang:** investigation (equal). **Huiyu Hu:** investigation (equal). **Jiawen Huang:** investigation (equal), visualization (equal), writing – review and editing (equal).

## Conflicts of Interest

The authors declare no conflicts of interest.

## Supporting information


**Data S1:** fsn370881‐sup‐0001‐DataS1.docx.

## Data Availability

Data available on request from the authors.
