# Supplementary material for: Citri Sarcodactylis Fructus Alleviates LPS‐Induced Acute Lung Injury by Inhibiting Inflammation and Inflammasome Activation
Source: Food Sci Nutr. 2025 Sep 1;13(9):e70881. doi: 10.1002/fsn3.70881 (PMC12400161; doi:10.1002/fsn3.70881)
Supplement: Supplementary file 1 — Data S1: fsn370881‐sup‐0001‐DataS1.docx. [file FSN3-13-e70881-s001.docx]

**Supplementary information**

| **Antibody** | **Source** | **Concentration** |
| --- | --- | --- |
| anti-IL6 | Affinity | 1:1000 |
| anti-TNF-α | Affinity | 1:1000 |
| anti-Phospho-IκBα (S32/S36) | Affinity | 1:1000 |
| Anti-IκBα | Affinity | 1:1000 |
| anti-COX2 | Affinity | 1:1000 |
| anti-iNOS | Affinity | 1:500 |
| anti-MCP-1 | Affinity | 1:1000 |
| anti-ICAM1 | Affinity | 1:1000 |
| anti-Phospho-NF-κB p65(S536) | Affinity | 1:1000 |
| anti-Caspase-8 | Affinity | 1:1000 |
| anti-NF-κB p65 | ImmunoWay | 1:1000 |
| anti-NLRP3 | ImmunoWay | 1:3000 |
| anti-ASC | ImmunoWay | 1:1000 |
| anti-Caspase-1 | ImmunoWay | 1:1000 |
| anti-NEK7 | ImmunoWay | 1:1000 |
| anti-IL1β | ImmunoWay | 1:1000 |
| anti-IL18 | ImmunoWay | 1:1000 |
| anti-GSDMD N-terminal | ImmunoWay | 1:1000 |
| anti-β-actin | ImmunoWay | 1:10000 |
| anti-TLR4 | Proteintech | 1:1500 |
| anti-MyD88 | Proteintech | 1:1000 |

**Supplementary Table 1**. Antibodies used in Western blot analysis.

**Supplementary Table 2**. Primers used in qPCR analysis.

| **Gene** | **Forward Primer (5' → 3')** | **Reward Primer (5' → 3')** |
| --- | --- | --- |
| *IL1α* | CGAAGACTACAGTTCTGCCATT | GACGTTTCAGAGGTTCTCAGAG |
| *IL1β* | CCGTGGACCTTCCAGGATGA | GGGAACGTCACACACCAGCA |
| *IL6* | TAGTCCTTCCTACCCCAATTTCC | TTGGTCCTTAGCCACTCCTTC |
| *TNF-α* | AGCCCCCAGTCTGTATCCTT | CTCCCTTTGCAGAACTCAGG |
| *Ccl3* | TTCTCTGTACCATGACACTCTGC | CGTGGAATCTTCCGGCTGTAG |
| *Ccl4* | TTCCTGCTGTTTCTCTTACACCT | CTGTCTGCCTCTTTTGGTCAG |
| *Ccl5* | TCGAGTGACAAACACGACTGC | GCTGCTTTGCCTACCTCTCC |
| *Ccl7* | GCTGCTTTCAGCATCCAAGTG | CCAGGGACACCGACTACTG |
| *Cxcl1* | CTGGGATTCACCTCAAGAACATC | CAGGGTCAAGGCAAGCCTC |
| *Cxcl9* | TCCTTTTGGGCATCATCTTCC | TTTGTAGTGGATCGTGCCTCG |
| *Cxcl10* | CCAAGTGCTGCCGTCATTTTC | GGCTCGCAGGGATGATTTCAA |
| *Cxcl11* | GGCTTCCTTATGTTCAAACAGGG | GCCGTTACTCGGGTAAATTACA |
| *TLR4* | TTTATTCAGAGCCGTTGGTG | CAGAGGATTGTCCTCCCATT |
| *MyD88* | TCATGTTCTCCATACCCTTGGT | AAACTGCGAGTGGGGTCAG |
| *COX2* | GTGGAAAAACCTCGTCCAGA | GCTCGGCTTCCAGTATTGAG |
| *iNOS* | CTCACTGGGACAGCACAGAA | GGCCTTGTGGTGAAGAGTGT |
| *MCP-1* | TTAAAAACCTGGATCGGAACCAA | GCATTAGCTTCAGATTTACGGGT |
| *ICAM1* | GTGATGCTCAGGTATCCATCCA | CACAGTTCTCAAAGCACAGCG |
| *F4/80* | CCCCAGTGTCCTTACAGAGTG | GTGCCCAGAGTGGATGTCT |
| *CD68* | TGTCTGATCTTGCTAGGACCG | GAGAGTAACGGCCTTTTTGTGA |
| *IL27* | CTGTTGCTGCTACCCTTGCTT | CACTCCTGGCAATCGAGATTC |
| *VCAM1* | AGTTGGGGATTCGGTTGTTCT | CCCCTCATTCCTTACCACCC |
| *NLRP3* | TGTGAGAAGCAGGTTCTACTCT | TGTAGCGACTGTTGAGGTCCA |
| *ASC* | CTTGTCAGGGGATGAACTCAAAA | GCCATACGACTCCAGATAGTAGC |
| *Caspase-1* | ACAAGGCACGGGACCTATG | TCCCAGTCAGTCCTGGAAATG |
| *NEK7* | GCTGTCTGCTATATGAGATGGC | CCGAATAGTGATCTGACGGGAG |
| *Caspase-8* | TGCTTGGACTACATCCCACAC | TGCAGTCTAGGAAGTTGACCA |
| *IL18* | GACTCTTGCGTCAACTTCAAGG | CAGGCTGTCTTTTGTCAACGA |
| *GSDMD* | CCATCGGCCTTTGAGAAAGTG | ACACATGAATAACGGGGTTTCC |
| *β-actin* | GGCTGTATTCCCCTCCATCG | CCAGTTGGTAACAATGCCATGT |

**Supplementary Table 3**. Ingredients from CSF were identified by UPLC-MS/MS analysis.

| **NO.** | **RT**  **(min)** | **Adducts** | **Formula** | **Mass Error**  **(ppm)** | **Identification** | **Fragment Ions** | **Observed**  **m/z** | **Ion mode** |
| --- | --- | --- | --- | --- | --- | --- | --- | --- |
| 1 | 0.96 | M-H2O-H, M-H | C_4_H_6_O_5_ | -0.94 | Malic acid | 71.0138, 72.9931, 89.0244, 115.0035, 133.0139 | 133.0141 | Negative |
| 2 | 1.21 | M-H | C_6_H_8_O_7_ | -0.14 | Citric acid | 85.0295, 87.0087, 111.0086, 129.0192, 173.009, 191.0195 | 191.0197 | Negative |
| 3 | 2.12 | M+H | C_6_H_8_O_4_ | -2.71 | 3,5-dihydroxy-6-methyl-2,3-dihydropyran-4-one | 69.0341, 71.0497, 81.0338, 85.0289, 99.0441, 109.0284, 127.0385, 145.0486 | 145.0491 | Positive |
| 4 | 3.48 | M-H | C_7_H_6_O_4_ | -0.89 | Protocatechuic acid | 109.0294, 153.0191 | 153.0192 | Negative |
| 5 | 4.23 | M+FA-H | C_16_H_18_O_9_ | -0.94 | Scopolin | 176.0113, 191.0347, 399.0905 | 399.0930 | Negative |
| 6 | 4.27 | M+H-H2O | C_10_H_10_O_5_ | -2.20 | 5-Hydroxyferulic acid | 105.0699, 133.028, 133.0644, 161.0592, 178.0253, 193.049 | 193.0491 | Positive |
| 7 | 4.66 | M-H, 2M-H, M+FA-H | C_16_H_22_O_9_ | -0.24 | Sweroside | 151.0763, 177.0556, 195.0661, 357.1188 | 357.1190 | Negative |
| 8 | 4.72 | M+H, M+Na | C_27_H_30_O_16_ | -3.43 | Rutin | 85.0287, 303.0485, 465.1009 | 611.1586 | Positive |
| 9 | 4.83 | M-H | C_21_H_20_O_10_ | -0.12 | Vitexin | 61.9883, 283.0607, 311.0561, 341.0666, 431.0977 | 431.0983 | Negative |
| 10 | 4.90 | M+NH4 | C_12_H_10_O_5_ | -3.71 | 2-Methyl-5-carboxymethyl-7-hydroxychromone | 91.0545, 120.0806, 134.0445, 146.0600, 188.0699, 192.0733, 206.0806, 216.0646, 234.0748, 252.0861 | 252.0858 | Positive |
| 11 | 5.07 | M-H | C_27_H_32_O_14_ | 0.23 | Naringin |  | 579.1721 | Negative |
| 12 | 5.13 | M+FA-H, 2M-H, M-H | C_28_H_34_O_15_ | 0.40 | Hesperidin | 286.0483, 301.0714, 609.1826 | 609.1827 | Negative |
| 13 | 5.16 | M+H, M+K, M+Na, M+NH4 | C_28_H_34_O_15_ | -3.96 | Neohesperidin | 177.0540, 195.0281, 263.0542, 303.0848, 345.0952, 369.0951, 413.1215, 431.1321, 449.1428, 465.1361 | 611.1946 | Positive |
| 14 | 5.46 | M-H | C_9_H_8_O_3_ | -0.15 | 2-Hydroxycinnamic acid | 119.0502, 163.04 | 163.0400 | Negative |
| 15 | 5.95 | M+H | C_11_H_6_O_4_ | -2.55 | Xanthotoxol | 147.0436, 175.0385, 203.0332 | 203.0333 | Positive |
| 16 | 6.12 | M-H | C_15_H_10_O_6_ | 0.06 | Luteolin | 285.0403 | 285.0405 | Negative |
| 17 | 6.29 | M-H | C_11_H_6_O_4_ | 0.01 | Bergaptol | 116.9283, 201.0193 | 201.0193 | Negative |
| 18 | 6.32 | M+H-H2O, M+H | C_22_H_26_O_8_ | -4.44 | Syringaresinol | 330.1086, 339.1205, 343.1161, 351.1205, 369.1338, 371.1474, 381.1068, 383.1469, 401.1559, 401.1724 | 401.1576 | Positive |
| 19 | 6.44 | M+H-H2O, M+H, M+NH4, M+K | C_17_H_18_O_7_ | -4.22 | Byakangelicin | 67.0547, 85.0651, 218.0202, 231.028, 233.0435, 317.1004 | 317.1006 | Positive |
| 20 | 6.75 | M+H | C_10_H_8_O_3_ | -2.98 | Herniarin | 121.0647, 133.0644, 145.0279, 149.0228, 177.0536 | 177.0541 | Positive |
| 21 | 6.83 | M-H | C_15_H_12_O_5_ | -0.02 | Naringenin | 119.0501, 151.0035, 271.0613 | 271.0612 | Negative |
| 22 | 7.10 | M-H | C_16_H_12_O_6_ | -0.50 | Diosmetin | 284.0326, 299.0558 | 299.0560 | Negative |
| 23 | 7.19 | M+H | C_16_H_14_O_6_ | -4.59 | Hesperetin | 153.0177, 177.054, 202.0252, 303.085 | 303.0849 | Positive |
| 24 | 8.03 | M+H, M+Na | C_12_H_8_O_4_ | -4.06 | Bergapten | 202.0254, 217.0489 | 217.0487 | Positive |
| 25 | 8.29 | M+H | C_16_H_14_O_5_ | -4.65 | Heraclenin | 203.0331, 287.0896 | 287.0901 | Positive |
| 26 | 8.51 | M+H, M+K, M+Na, M+H-H2O | C_26_H_30_O_8_ | -3.70 | Limonin | 339.1951, 367.1899, 393.2084, 407.1859, 409.1976, 411.2178, 425.1945, 427.2102, 471.1933, 471.1994 | 471.1996 | Positive |
| 27 | 8.99 | M+FA-H | C_16_H_26_O_3_ | -0.66 | 4-methoxy-5-hydroxybisabola-2,10-diene-9-one | 174.9559, 267.1965, 293.176, 311.1872 | 311.1862 | Negative |
| 28 | 9.21 | M+H | C_18_H_28_O_3_ | -4.74 | 15,16-bisnor-13-oxo-8(17)-labden-19-oicacid | 81.0702, 93.0702, 107.0855, 134.0597, 147.1164, 149.0959, 257.1903, 275.1998, 293.1510, 293.2110 | 293.2097 | Positive |
| 29 | 9.31 | M-H | C_14_H_14_O_3_ | -0.68 | Demethyl-suberosin | 174.032, 206.0219, 229.0868 | 229.0869 | Negative |
| 30 | 9.68 | M+Na | C_20_H_20_O_7_ | -4.23 | Tangeretin |  | 395.1085 | Positive |
| 31 | 9.75 | M+FA-H, 2M-H | C_26_H_30_O_7_ | 0.28 | Obacunone | 207.9405, 453.1921, 499.1985 | 499.1975 | Negative |

**Supplementary Table 4**. Molecular docking details.

| **Target-compound** | **2D image** | **Binding energies** | **Binding details** |
| --- | --- | --- | --- |
| NF κB p65 -Bergapten | 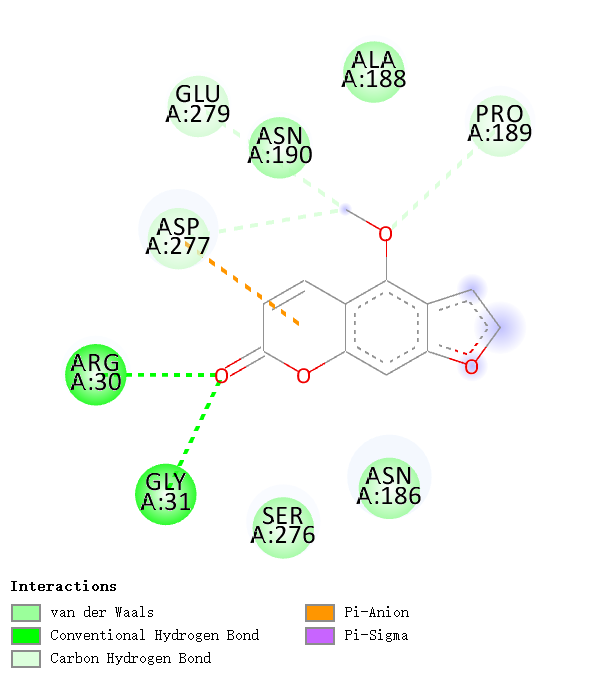 | -6.2kcal/mol | The binding mode involves bergapten forming hydrogen bonds with protein residues GLY-31 and ARG-30, C-H bonds with residues GLU-279, ASP-277, and PRO-189, and van der Waals forces with residues ALA-188, SER-276, and ASN-186. Additionally, it undergoes Pi-Anion interaction with residue ASP-277 and Pi-Sigma interaction with residue ASP-277. |
| NF κB p65 -Bergaptol | 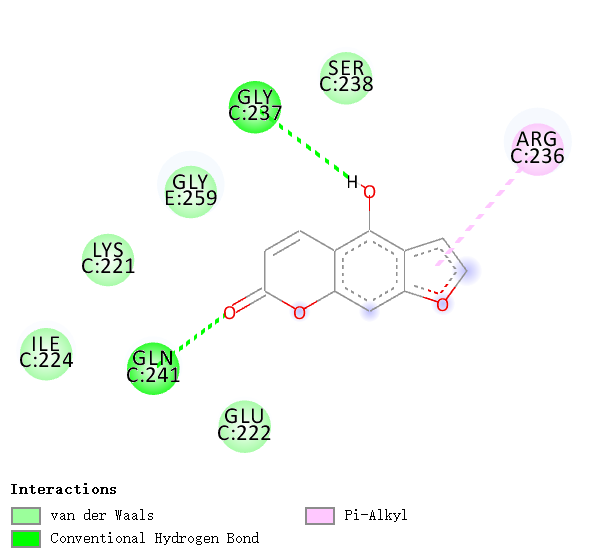 | -6.7kcal/mol | The binding mode involves bergaptol forming hydrogen bonds with protein residues GLN-241, LYS-221, and PHE-239, van der Waals forces with residues ILE-224, LYS-221, GLY-259, SER-238, and GLU-222, and Pi-Alkyl hydrophobic interactions with residue ARG-236. |
| NF κB p65 -Byakangelicin | 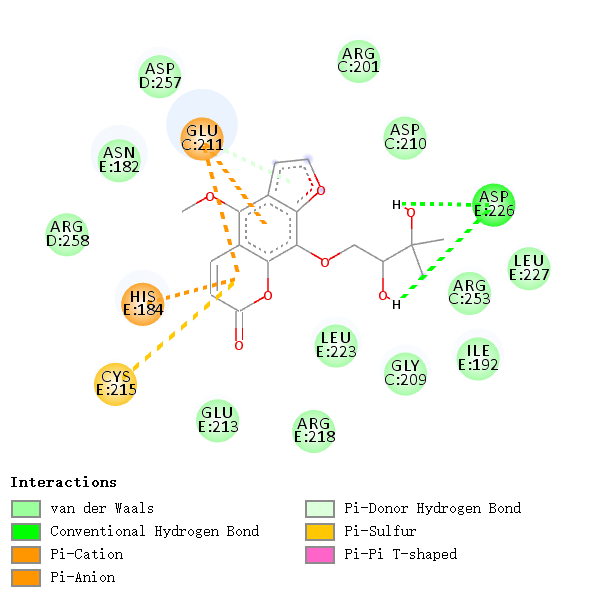 | -7.2kcal/mol | The binding mode involves byakangelicin forming a hydrogen bond with protein residue ASP-226, van der Waals forces with residues ARG-258, ASN-182, ASP-257, ARG-201, ASP-210, LEU-227, ARG-253, ILE-192, GLY-209, LEU-223, ARG-218, and GLU-213, a Pi-Cation interaction with residue HIS-184, a Pi-Anion interaction with residue GLU-211, a Pi-Donor Hydrogen Bond interaction with residues GLU-211 and HIS-184, a Pi-Sulfur interaction with residue CYS-215, and a Pi-Pi T-shaped interaction with residue HIS-184. |
| NF κB p65 -Heraclenin | 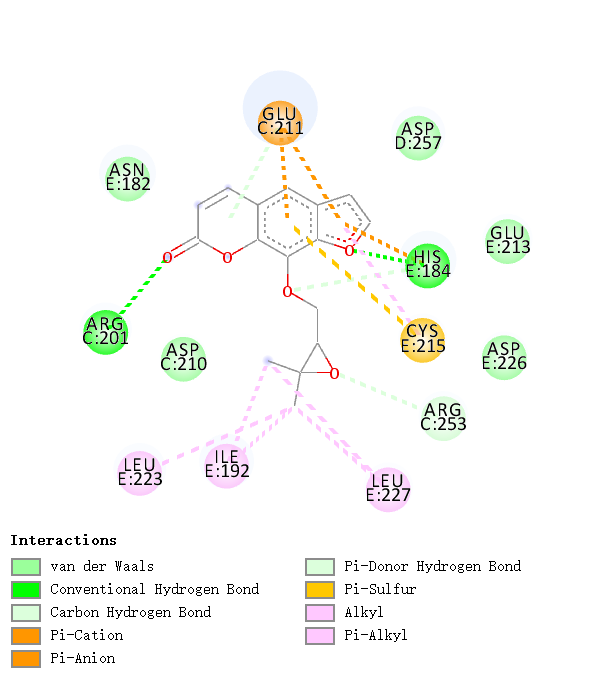 | -7.4kcal/mol | The binding mode involves heraclenin forming hydrogen bonds with protein residues ARG-201 and HIS-184, van der Waals forces with residues ASN-182, ASP-257, GLU-213, ASP-226, and ASP-210, and C-H bonds with residues HIS-184 and ARG-253. Additionally, it undergoes a Pi-Cation interaction with residue HIS-184, a Pi-Anion interaction with residue GLU-211, a Pi-Donor Hydrogen Bond interaction with residues GLU-211 and HIS-184, and a Pi-Sulfur interaction with residue CYS-215. It also forms Alkyl hydrophobic interactions with residues LEU-223, ILE-192, and LEU-227, as well as Pi-Alkyl hydrophobic interactions with residue CYS-215. |
| NF κB p65 -Hesperetin | 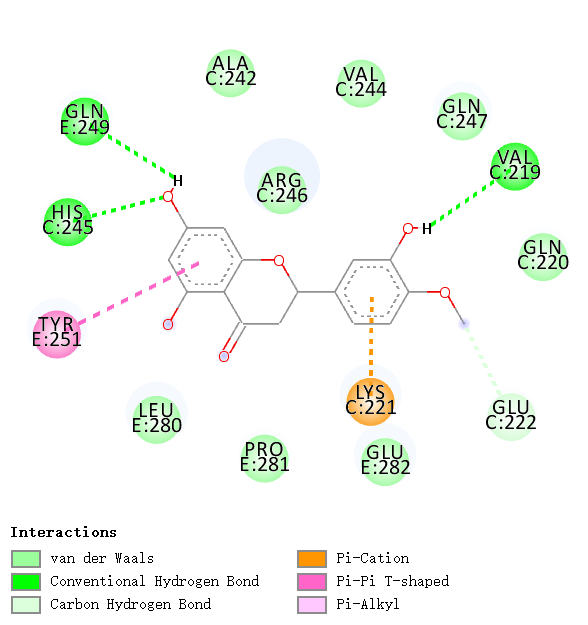 | -8.7kcal/mol | The binding mode involves hesperetin forming hydrogen bonds with protein residues GLN-249, HIS-245, and VAL-219, van der Waals forces with residues ALA-242, VAL-244, ARG-246, GLN-247, GLN-220, GLU-282, PRO-281, and LEU-280, and a C-H bond with residue GLU-222. It also undergoes a Pi-Cation interaction with residue LYS-221, a Pi-Pi T-Shaped interaction with residue TYR-251, a Pi-Sulfur interaction with residue CYS-215, and a Pi-Alkyl hydrophobic interaction with residue LYS-221. |
| NF κB p65 -Hesperidin | 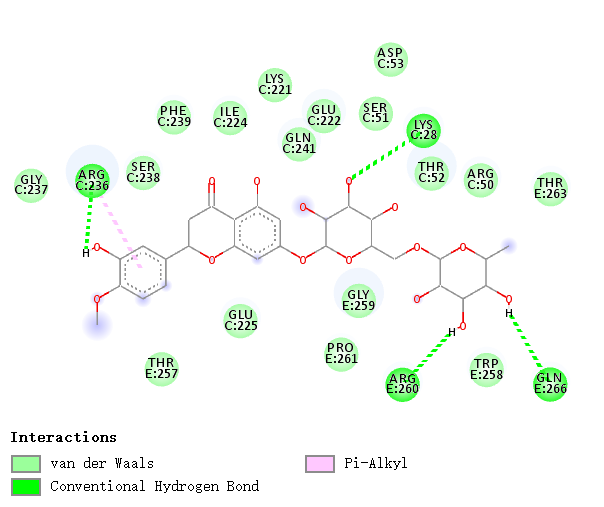 | -9.5kcal/mol | The binding mode involves hesperidin forming hydrogen bonds with protein residues ARG-236 (twice), GLN-241, GLY-259, and GLN-265, van der Waals forces with residues GLY-237, SER-238, PHE-239, ILE-224, LYS-221, GLN-241, GLU-222, SER-51, ASP-53, THR-52, ARG-50, THR-263, TRP-258, GLY-259, PRO-261, GLU-225, and THR-257, and a Pi-Alkyl hydrophobic interaction with residue ARG-236. |
| NF κB p65 -Limonin | 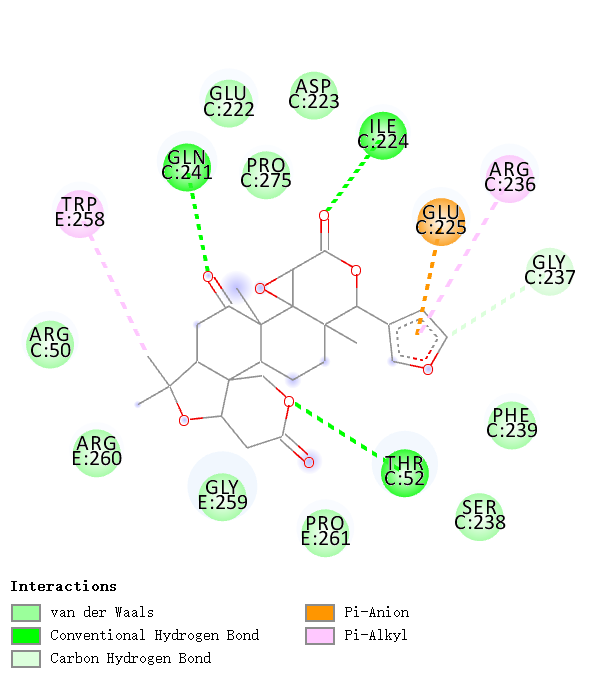 | -9.6kcal/mol | The binding mode involves limonin forming hydrogen bonds with protein residues GLN-241 and ILE-224, van der Waals forces with residues ARG-50, ARG-260, GLY-259, PRO-261, SER-238, PHE-239, GLU-222, ASP-223, and PRO-275, and a C-H bond with residue GLY-237. It also undergoes a Pi-Anion interaction with residue GLU-225 and Pi-Alkyl hydrophobic interactions with residues TRP-258 and ARG-236. |
| NF κB p65 -Naringenin | 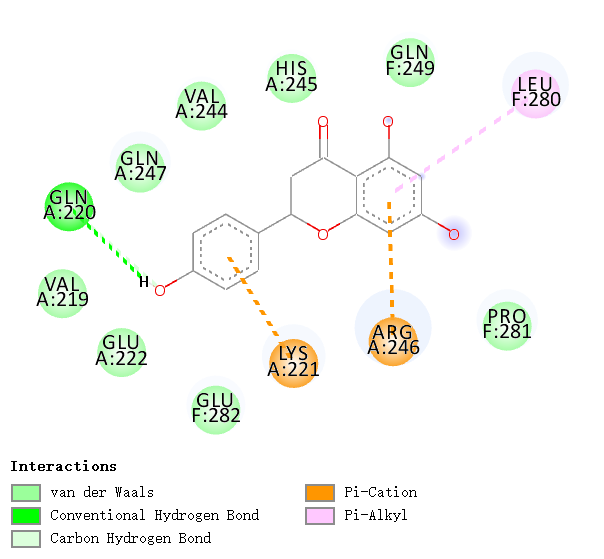 | -8.0kcal/mol | The binding mode involves naringenin forming hydrogen bonds with protein residues GLN-220 and TYR-251, van der Waals forces with residues VAL-219, GLU-222, GLU-282, PRO-281, GLN-247, VAL-244, HIS-245, and GLN-249, and a C-H bond with residue GLN-220. It also undergoes Pi-Cation interactions with residues LYS-221 and ARG-246, and Pi-Alkyl hydrophobic interactions with residues LEU-280 and LYS-221. |
| NF κB p65 -Naringin | 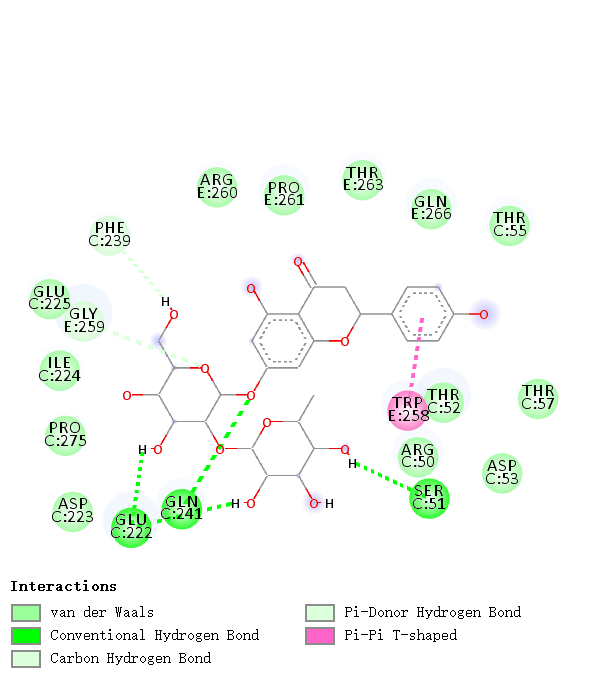 | -8.9kcal/mol | The binding mode involves naringin forming hydrogen bonds with protein residues GLU-222, GLN-241, SER-51, and LYS-28, van der Waals forces with residues ASP-223, PRO-275, ILE-224, GLU-225, ARG-260, PRO-261, THR-263, GLN-266, THR-55, THR-57, ASP-53, and ARG-50, and a C-H bond with residue GLY-259. It also undergoes a Pi-Donor Hydrogen Bond interaction with residue PHE-239 and a Pi-Pi T-shaped interaction with residue TRP-258. |
| NF κB p65-Neohesperidin | 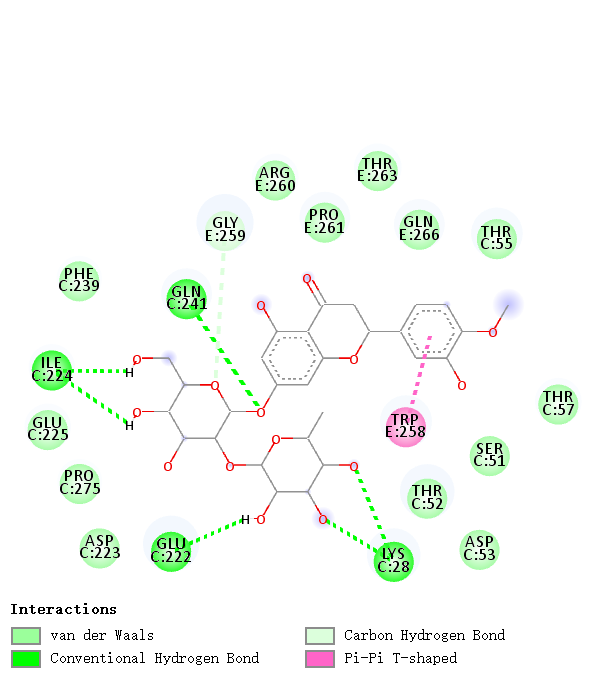 | -9.0kcal/mol | The binding mode involves neohesperidin forming hydrogen bonds with protein residues ILE-224, GLU-222, LYS-28, and GLN-241, van der Waals forces with residues ASP-223, PRO-275, GLU-225, PHE-239, ARG-260, PRO-261, THR-263, GLN-266, THR-55, THR-57, SER-51, THR-52, and ASP-53, and a C-H bond with residue GLY-259. It also undergoes a Pi-Pi T-shaped interaction with residue TRP-258. |
| NF κB p65-Obacunone | 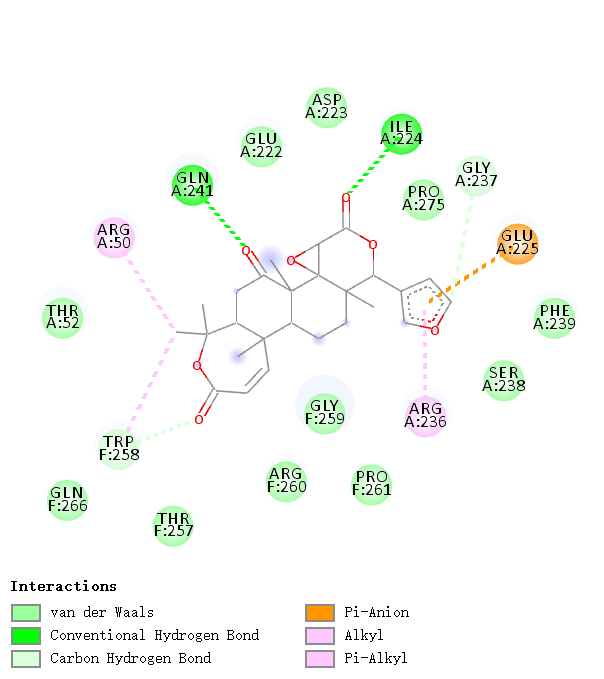 | -9.4kcal/mol | The binding mode involves obacunone forming hydrogen bonds with protein residues GLN-241 and ILE-224, van der Waals forces with residues THR-52, GLU-222, ASP-223, PRO-275, PHE-239, SER-238, PRO-261, ARG-260, GLY-259, THR-257, and GLN-266, and C-H bonds with residues GLY-237 and TRP-258. It also undergoes a Pi-Anion interaction with residue GLU-225, forms Alkyl hydrophobic interactions with residue ARG-50, and forms Pi-Alkyl hydrophobic interactions with residues TRP-258 and ARG-236. |
| NF κB p65-Rutin | 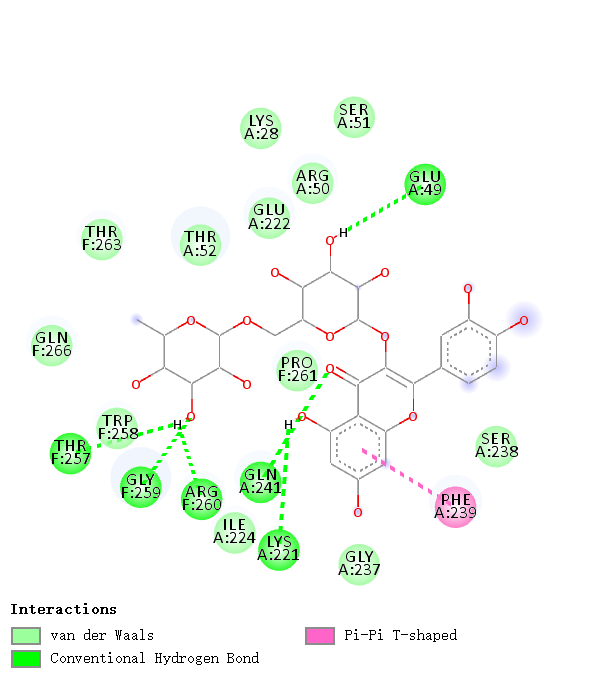 | -8.8kcal/mol | The binding mode involves rutin forming hydrogen bonds with protein residues THR-257, GLY-259, ARG-260, LYS-221, GLN-241, GLU-49, GLU-222, and PHE-239, and van der Waals forces with residues TRP-258, GLN-266, THR-263, THR-52, GLU-222, ARG-50, LYS-28, SER-51, SER-238, GLY-237, PRO-261, and ILE-224. It also undergoes a Pi-Pi T-shaped interaction with residue PHE-239. |
| NF κB p65-Syringaresinol | 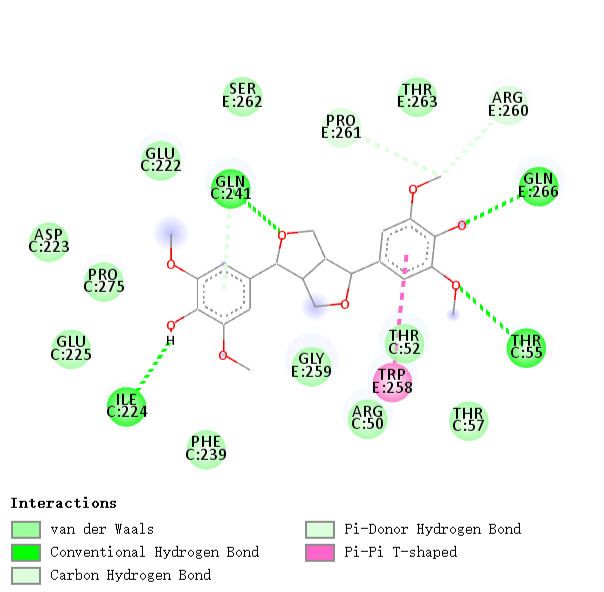 | -7.5kcal/mol | The binding mode involves syringaresinol forming hydrogen bonds with protein residues ILE-224, GLN-241, and GLN-266, van der Waals forces with residues PHE-239, GLY-259, ARG-50, THR-57, THR-52, THR-263, SER-262, GLU-222, ASP-223, PRO-275, and GLU-225, and C-H bonds with residues PRO-261 and ARG-260. It also undergoes a Pi-Donor Hydrogen Bond interaction with residue GLN-241 and a Pi-Pi T-shaped interaction with residue TRP-258. |
| NF κB p65-Tangeretin | 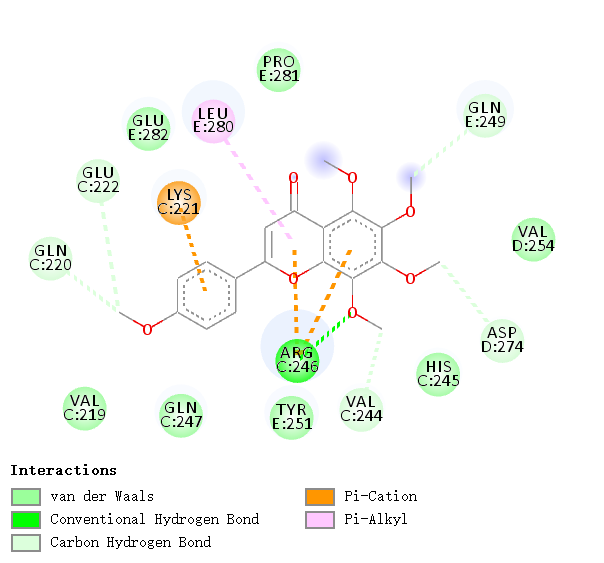 | -7.8kcal/mol | The binding mode involves tangeretin forming hydrogen bonds with protein residues ARG-246, HIS-245, and LYS-221, van der Waals forces with residues VAL-219, GLN-247, TYR-251, HIS-245, VAL-254, PRO-281, and GLU-282, and C-H bonds with residues GLU-222, GLN-220, VAL-244, ASP-274, and GLN-249. It also undergoes Pi-Cation interactions with residues LYS-221 and ARG-246, and Pi-Alkyl hydrophobic interactions with residues LEU-280 and LYS-221. |
| NF κB p65-vitexin | 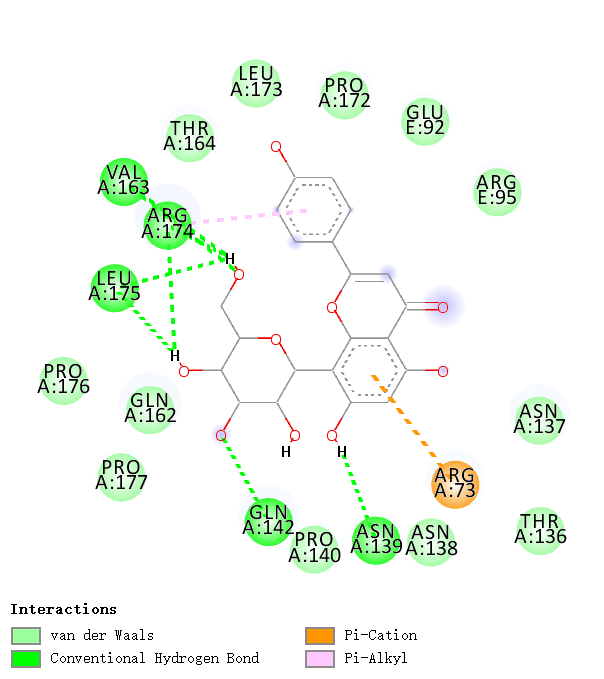 | -8.8kcal/mol | The binding mode involves vitexin forming hydrogen bonds with protein residues LEU-175, ARG-174, GLN-142, and ASN-139, van der Waals forces with residues PRO-176, GLN-162, PRO-177, PRO-140, ASN-138, THR-136, ASN-137, ARG-95, GLU-92, PRO-172, LEU-173, and THR-164. It also undergoes a Pi-Cation interaction with residue ARG-73 and a Pi-Alkyl hydrophobic interaction with residue ARG-174. |
| NF κB p65-Xanthotoxol | 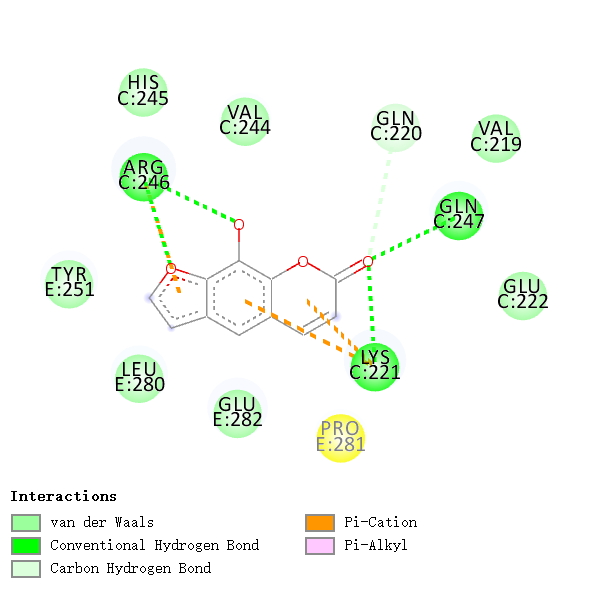 | -7.4kcal/mol | The binding mode involves xanthotoxol forming hydrogen bonds with protein residues ARG-246, GLN-247, VAL-219, and GLN-220, van der Waals forces with residues GLU-282, LEU-280, TYR-251, HIS-245, VAL-244, VAL-219, and GLU-222, and a C-H bond with residue GLN-220. It also undergoes Pi-Cation interactions with residues LYS-221 and ARG-246, and a Pi-Alkyl hydrophobic interaction with residue LYS-221. |
| NLRP3-Bergapten | 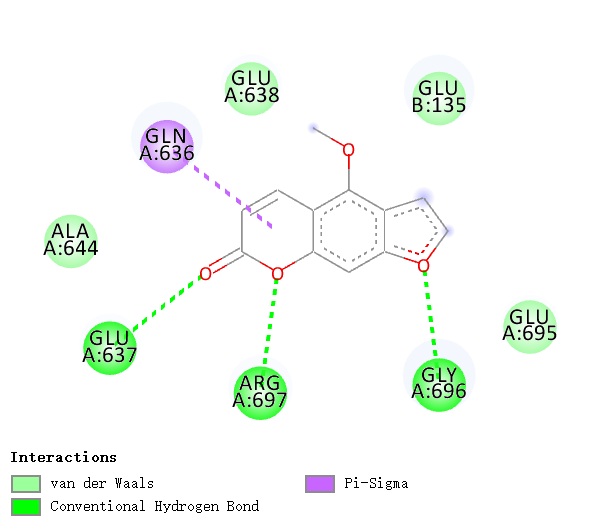 | -6.9kcal/mol | The binding mode involves bergapten forming hydrogen bonds with protein residues GLU-637, ARG-697, and GLY-696, van der Waals forces with residues ALA-644, GLU-638, GLU-135, and GLU-695, and undergoing a Pi-Sigma interaction with residue GLN-636. |
| NLRP3-Bergaptol | 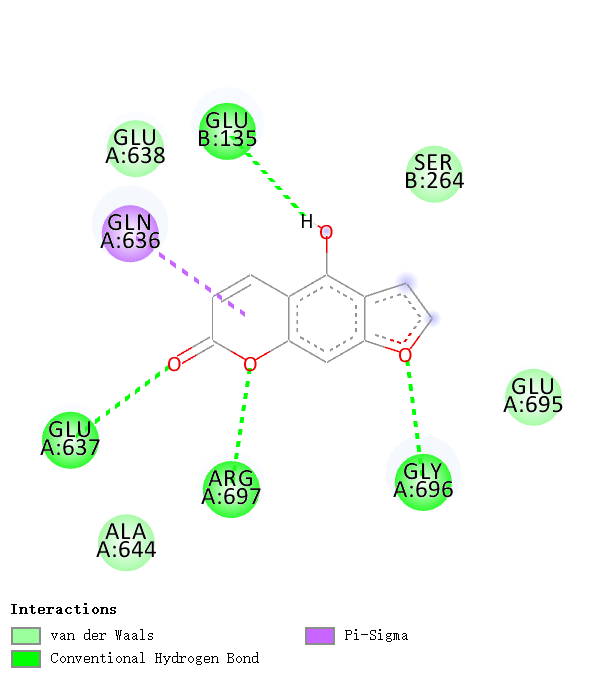 | -7.0kcal/mol | The binding mode involves bergaptol forming hydrogen bonds with protein residues GLU-637, ARG-697, GLY-696, and GLU-135, van der Waals forces with residues ALA-644, GLU-695, SER-264, and GLU-638, and undergoing a Pi-Sigma interaction with residue GLN-636. |
| NLRP3-Byakangelicin | 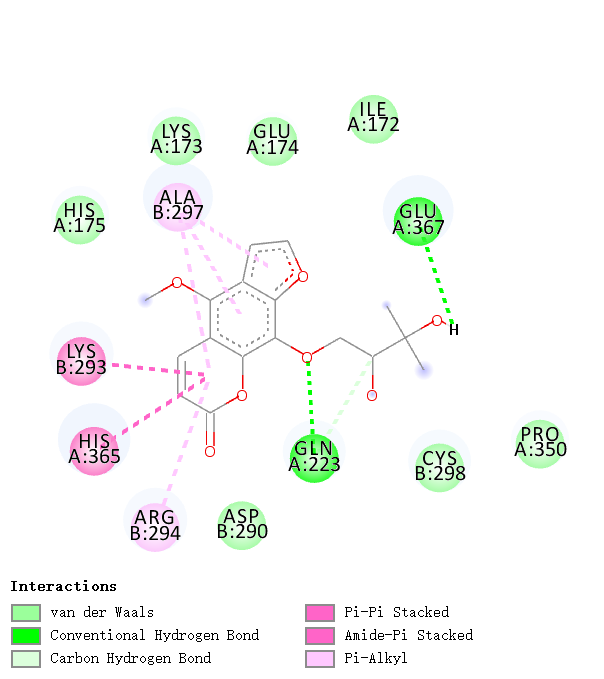 | -7.8kcal/mol | The binding mode involves byakangelicin forming hydrogen bonds with protein residues GLU-367, GLN-223, and ASP-290, van der Waals forces with residues HIS-175, LYS-173, GLU-174, ILE-172, PRO-350, CYS-298, and ASP-290, and a C-H bond with residue GLN-223. It also undergoes a Pi-Pi Stacked interaction with residue HIS-365, Amide-Pi Stacked interactions with residues LYS-293 and ARG-294, and forms Pi-Alkyl hydrophobic interactions with residues ALA-297, LYS-293, and ARG-294. |
| NLRP3-Heraclenin | 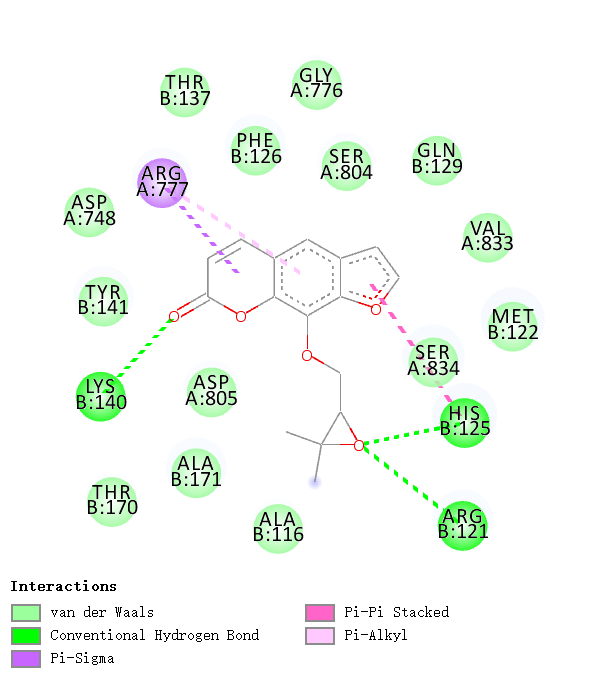 | -7.3kcal/mol | The binding mode involves heraclenin forming hydrogen bonds with protein residues HIS-125, ARG-121, ASP-748, LYS-140, and TYR-141, van der Waals forces with residues THR-170, ALA-171, ALA-116, ASP-805, SER-834, MET-122, VAL-833, GLN-129, SER-804, GLY-776, PHE-126, THR-137, ASP-748, and TYR-141. It also undergoes a Pi-Sigma interaction with residue ARG-777, a Pi-Pi Stacked interaction with residue HIS-125, and forms a Pi-Alkyl hydrophobic interaction with residue ARG-777. |
| NLRP3-Hesperetin | 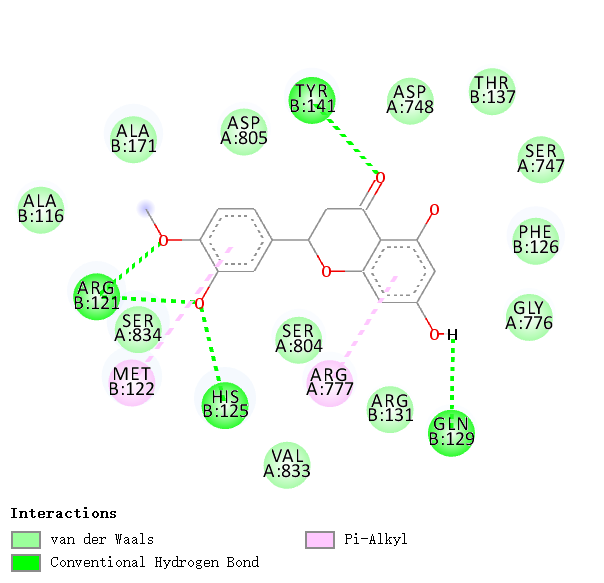 | -8.4kcal/mol | The binding mode involves hesperetin forming hydrogen bonds with protein residues ARG-121, HIS-125, GLN-129, TYR-141, and ARG-131, van der Waals forces with residues VAL-833, ARG-31, GLY-776, PHE-126, SER-747, THR-137, ASP-748, ASP-805, ALA-171, ALA-116, SER-834, and SER-804, and forms Pi-Alkyl hydrophobic interactions with residues ARG-777 and MET-122. |
| NLRP3-Hesperidin | 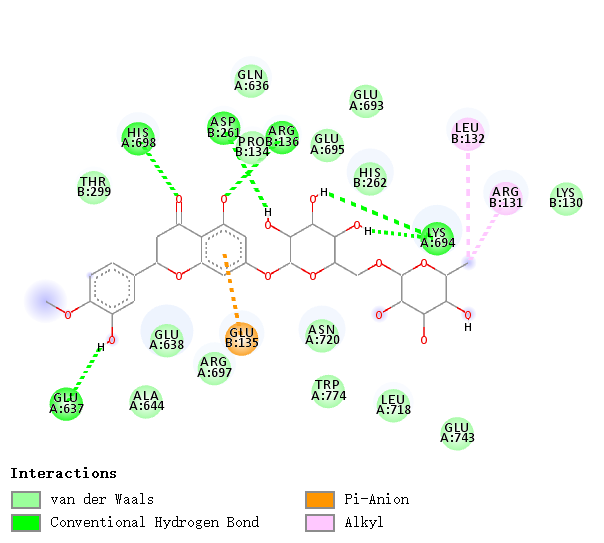 | -10.3kcal/mol | The binding mode involves hesperidin forming hydrogen bonds with protein residues HIS-698, LYS-694, GLU-637, ASP-261, GLU-135, and ARG-136, van der Waals forces with residues THR-299, GLU-638, ALA-644, ARG-697, ASN-720, TRP-774, LEU-718, GLU-743, LYS-130, GLU-693, GLU-695, HIS-262, GLN-636, and PRO-134. It also undergoes a Pi-Anion interaction with residue GLU-135 and forms Alkyl hydrophobic interactions with residues LEU-132 and ARG-131. |
| NLRP3-Limonin | 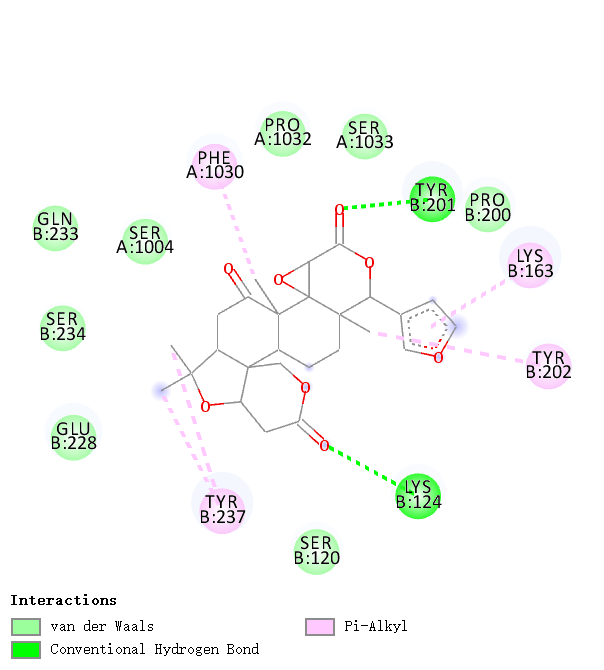 | -9.7kcal/mol | The binding mode involves limonin forming hydrogen bonds with protein residues LYS-124 and TYR-201, van der Waals forces with residues SER-120, GLU-228, SER-234, GLN-233, SER-1004, PRO-1032, SER-1033, and PRO-200, and Pi-Alkyl hydrophobic interactions with residues PHE-1030, LYS-163, TYR-202, and TYR-237. |
| NLRP3-Naringenin | 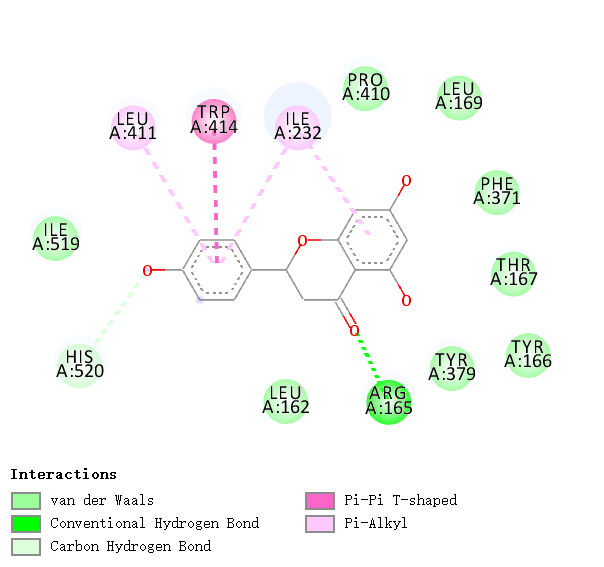 | -8.5kcal/mol | The binding mode involves naringenin forming a hydrogen bond with protein residue ARG-165, van der Waals forces with residues ILE-519, LEU-162, TYR-379, TYR-166, THR-167, PHE-371, LEU-169, and PRO-410, and a C-H bond with residue HIS-520. It also undergoes a Pi-Pi T-Shaped interaction with residue TRP-414 and forms Pi-Alkyl hydrophobic interactions with residues ILE-232 and LEU-411. |
| NLRP3-Naringin | 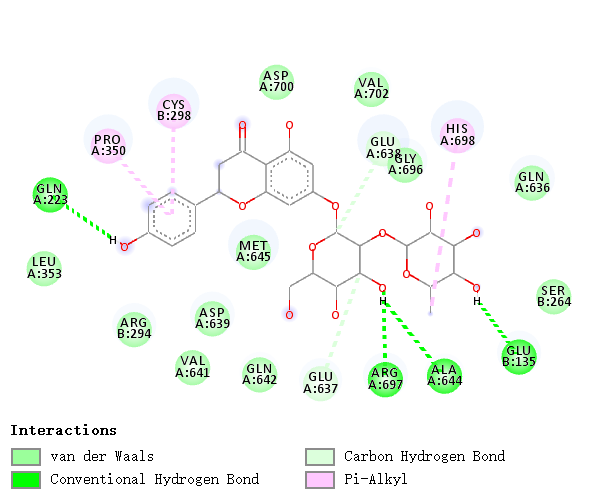 | -8.9kcal/mol | The binding mode involves naringin forming hydrogen bonds with protein residues ARG-697, ALA-644, GLU-135, and GLN-223, van der Waals forces with residues LEU-353, ARG-294, ASP-639, MET-645, VAL-641, GLN-642, SER-264, GLN-636, GLY-696, VAL-702, and ASP-700, and C-H bonds with residues GLU-637 and GLU-638. It also undergoes Pi-Alkyl hydrophobic interactions with residues HIS-698, CYS-298, and PRO-350. |
| NLRP3-Neohesperidin | 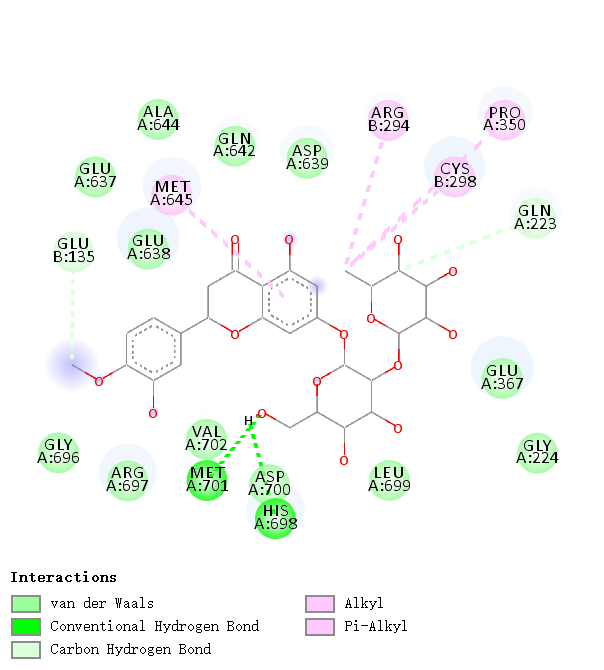 | -9.4kcal/mol | The binding mode involves neohesperidin forming hydrogen bonds with protein residues MET-701, VAL-702, ARG-697, HIS-698, and ASP-700, van der Waals forces with residues GLY-696, ARG-697, VAL-702, ASP-700, LEU-699, GLU-367, GLY-224, ASP-639, GLN-642, ALA-644, GLU-638, and GLU-637, and C-H bonds with residues GLU-135 and GLN-223. It also forms Alkyl hydrophobic interactions with residues ARG-294, PRO-350, and CYS-298, and Pi-Alkyl hydrophobic interactions with residue MET-645. |
| NLRP3-Obacunone | 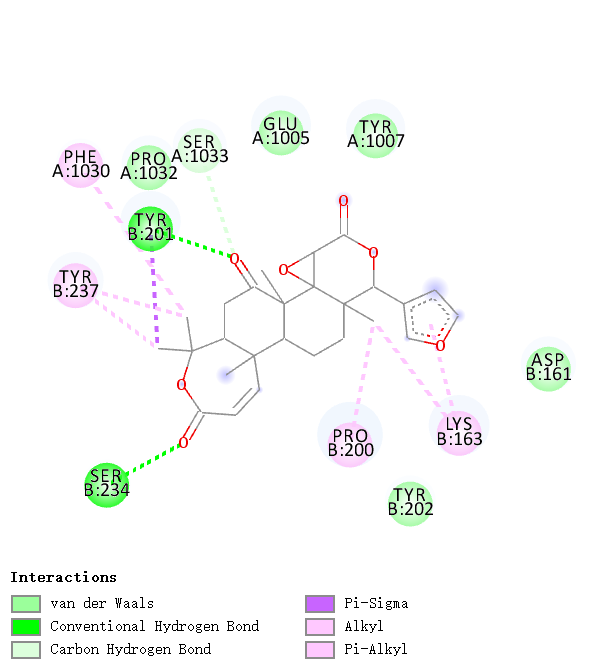 | -9.8kcal/mol | The binding mode involves obacunone forming hydrogen bonds with protein residues TYR-201 and SER-234, van der Waals forces with residues TYR-202, ASP-161, TYR-1007, GLU-1005, and PRO-1032, and a C-H bond with residue SER-1033. It also undergoes a Pi-Sigma interaction with residue TYR-201, forms Alkyl hydrophobic interactions with residues PRO-200 and LYS-163, and forms Pi-Alkyl hydrophobic interactions with residues PHE-1030, TYR-237, and LYS-163. |
| NLRP3-Rutin | 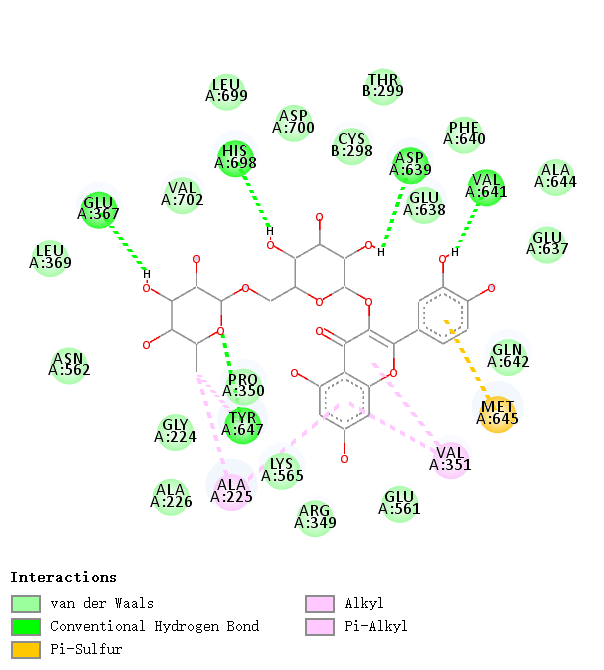 | -9.0kcal/mol | The binding mode involves rutin forming hydrogen bonds with protein residues LYS-565, ARG-349, GLU-367, VAL-641, and HIS-698, van der Waals forces with residues LEU-369, ASN-562, GLY-224, ALA-226, PRO-350, LYS-565, ARG-349, GLU-561, GLN-642, GLU-637, ALA-644, GLU-638, PHE-640, THR-299, CYS-298, ASP-700, LEU-699, and VAL-702. It also undergoes a Pi-Sulfur interaction with residue MET-645, forms Alkyl hydrophobic interactions with residue ALA-225, and forms Pi-Alkyl hydrophobic interactions with residues ALA-225, TYR-647, and VAL-351. |
| NLRP3-Syringaresinol | 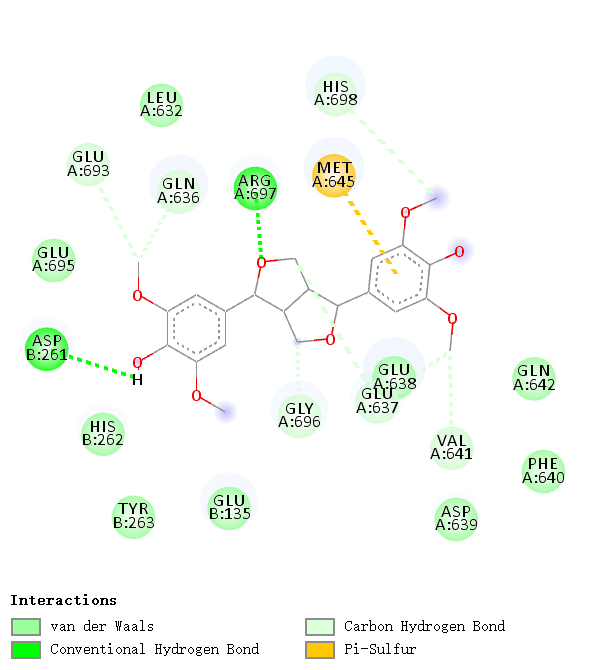 | -8.8kcal/mol | The binding mode involves syringaresinol forming hydrogen bonds with protein residues ARG-697 and ASP-261, van der Waals forces with residues HIS-262, TYR-263, GLU-135, GLU-638, ASP-639, PHE-640, GLN-642, LEU-632, and GLU-695, and C-H bonds with residues GLU-693, GLN-636, HIS-698, VAL-641, GLU-637, and GLY-696. It also undergoes a Pi-Sulfur interaction with residue MET-645. |
| NLRP3-Tangeretin | 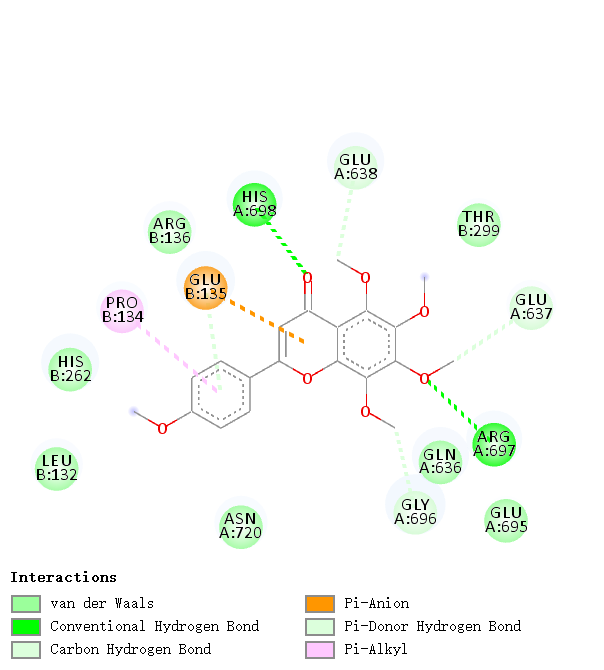 | -8.0kcal/mol | The binding mode involves tangeretin forming hydrogen bonds with protein residues HIS-698 and ARG-697, van der Waals forces with residues LEU-132, HIS-262, ARG-136, THR-299, GLU-695, ASN-720, and GLN-636, and C-H bonds with residues GLU-638, GLU-637, and GLY-696. It also undergoes a Pi-Anion interaction with residue GLU-135, a Pi-Donor Hydrogen Bond interaction with residue GLU-135, and forms a Pi-Alkyl hydrophobic interaction with residue PRO-134. |
| NLRP3-vitexin | 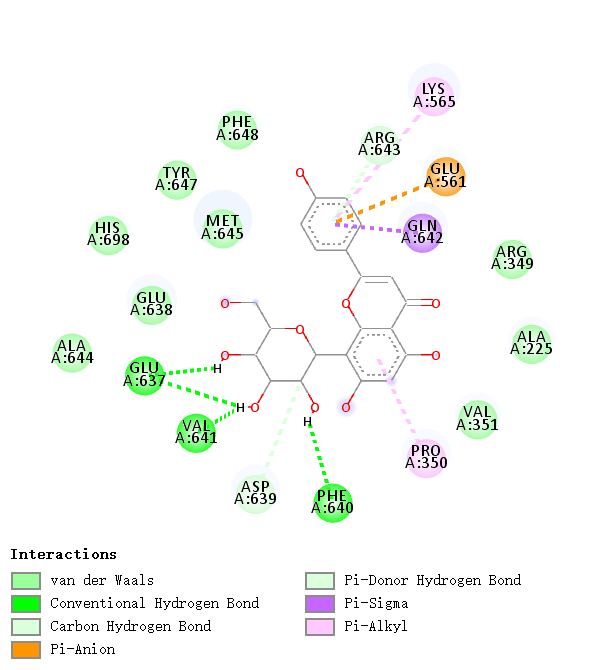 | -8.7kcal/mol | The binding mode involves vitexin forming hydrogen bonds with protein residues GLU-637, VAL-641, and PHE-640, van der Waals forces with residues ALA-644, GLU-638, HIS-698, MET-645, TYR-647, PHE-648, ARG-349, ALA-225, and VAL-351, and a C-H bond with residue ASP-639. It also undergoes a Pi-Anion interaction with residue GLU-561, a Pi-Donor Hydrogen Bond interaction with residue ARG-643, a Pi-Sigma interaction with residue GLN-642, and forms Pi-Alkyl hydrophobic interactions with residues PRO-350 and LYS-565. |
| NLRP3-Xanthotoxol | 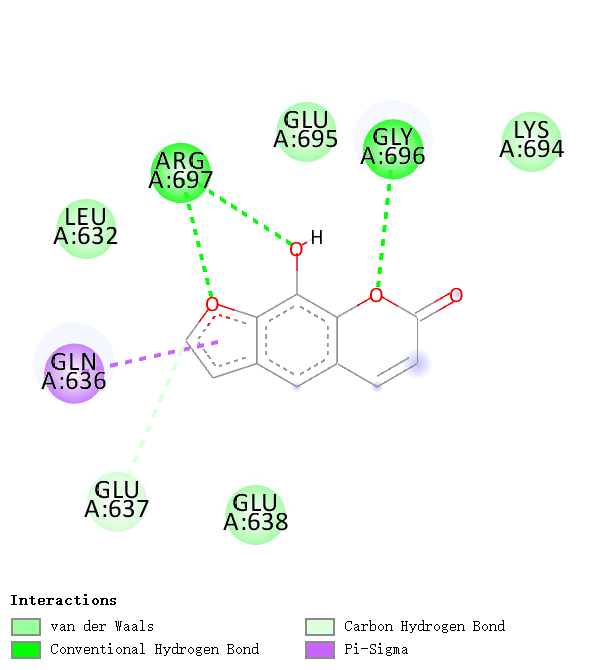 | -7.6kcal/mol | The binding mode involves xanthotoxol forming hydrogen bonds with protein residues ARG-697 and GLY-696, van der Waals forces with residues GLU-638, LEU-632, GLU-695, and LYS-694, and a C-H bond with residue GLU-637. It also undergoes a Pi-Sigma interaction with residue GLN-636. |

**Supplementary Table 5**. The binding energy by MMGBSA (kJ/mol).

| Type | NFκB p65-Bergapten | NFκB p65-Hesperetin | NFκB p65-Hesperidin | NFκB p65-Naringin | NLRP3-Bergapten | NLRP3-Hesperetin | NLRP3-Hesperidin | NLRP3-Naringin |
| --- | --- | --- | --- | --- | --- | --- | --- | --- |
| *E_VDW_* | -26.03±0.05 | -30.35±0.26 | -41.07±1.06 | -40.57±1.58 | -37.66±0.83 | -43.33±0.96 | -69.18±0.13 | -59.82±0.16 |
| *E_ELE_* | -8.26±0.55 | -22.55±4.86 | -26.15±0.32 | -53.45±1.4 | -52.07±0.79 | -34.91±2.35 | -74.61±3.1 | -50.3±5.65 |
| *E_GB_* | 16.62±0.53 | 38.19±0.88 | 43.3±3.21 | 71.73±0.6 | 54.63±0.38 | 51.73±0.33 | 104.65±0.34 | 68.99±1.29 |
| *E_SA_* | -2.51±0 | -3.01±0.02 | -4.12±0.17 | -3.79±0.02 | -2.48±0.02 | -3.73±0.03 | -6.62±0.02 | -5.81±0.01 |
| *G_binding energy_* | -20.18±0.77 | -17.72±4.95 | -28.04±3.4 | -26.08±2.2 | -37.57±1.21 | -30.24±2.56 | -45.75±3.13 | -46.94±5.79 |

*E_VDW_*: van der Waals energy

*E_ELE_*: eletrostatic energy

*E_GB_*: polar contribution to solvation

*E_SA_*: non-polar contribution to solvation

**
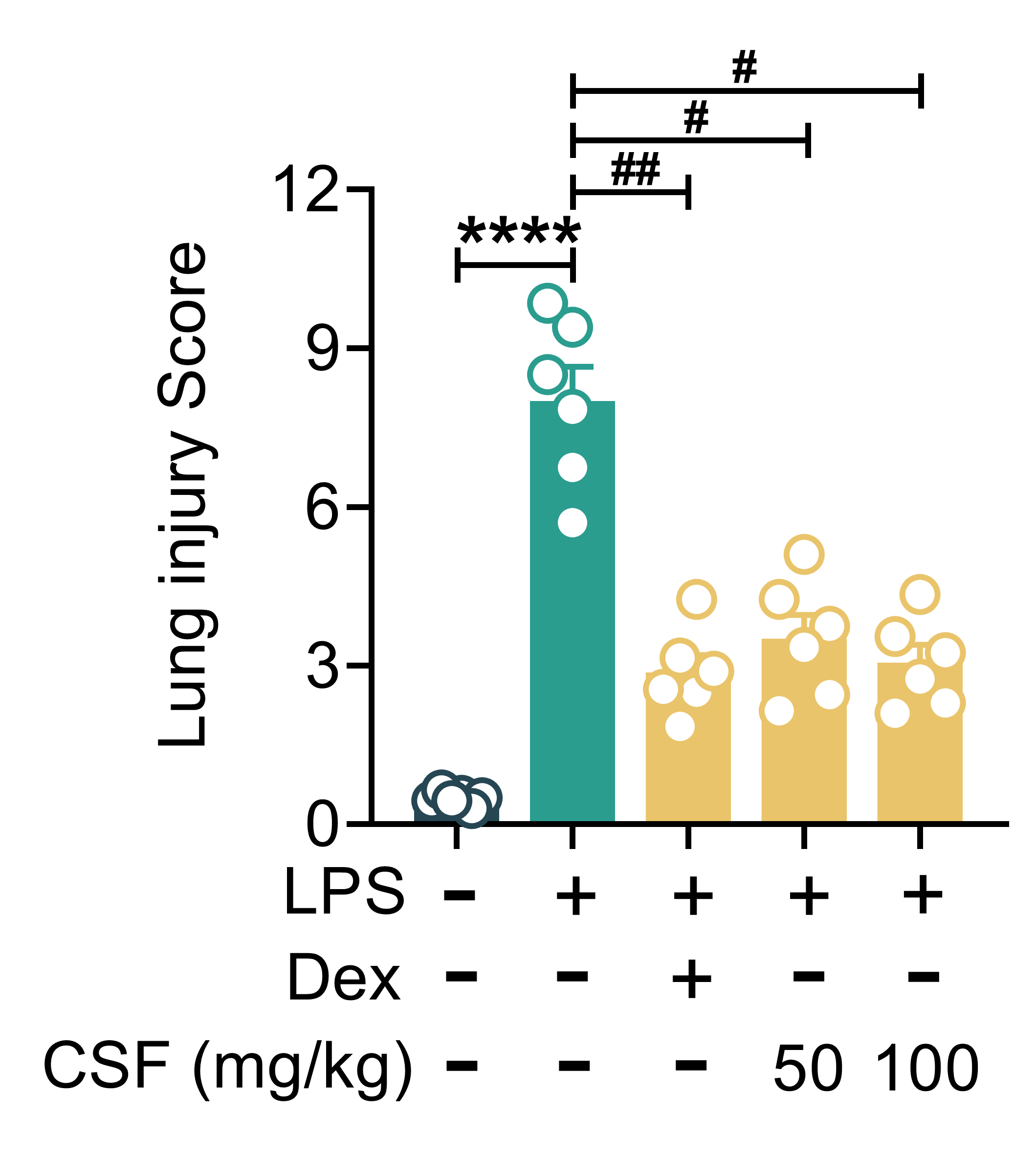
**

**Supplementary Figure 1**. The degree of lung damage. Each item was assessed using a 5-point scale (0-4 points): 0 points (normal: no injury); 1 point (mild injury: injury involving 25% of the visual field); 2 points (moderate injury: injury involving 50% of the visual field); 3 points (severe injury: injury involving 75% of the visual field); and 4 points (very severe injury: diffuse injury). The total lung injury score (ranging from 0 to 16 points) was calculated by summing the scores of these four indicators. Data are presented as mean ± SEM. ^****^*p* < 0.0001 vs. the control group. ^#^*p* < 0.05, ^##^*p* < 0.01 vs. the LPS group.
